# Multilevel-Regulated Metal–Organic Framework Platform Integrating Pore Space Partition and Open-Metal Sites for Enhanced CO_2_ Photoreduction to CO with Nearly 100% Selectivity

**DOI:** 10.1021/jacs.3c10090

**Published:** 2023-12-06

**Authors:** Hui-Li Zheng, Jian-Qiang Zhao, Ya-Yong Sun, An-An Zhang, Yu-Jia Cheng, Liang He, Xianhui Bu, Jian Zhang, Qipu Lin

**Affiliations:** † State Key Laboratory of Structural Chemistry, Fujian Institute of Research on the Structure of Matter, Chinese Academy of Sciences, Fuzhou 350002, China; ‡ University of Chinese Academy of Sciences, Beijing 100049, China; § Department of Chemistry and Biochemistry, California State University, Long Beach, California 90840, United States

## Abstract

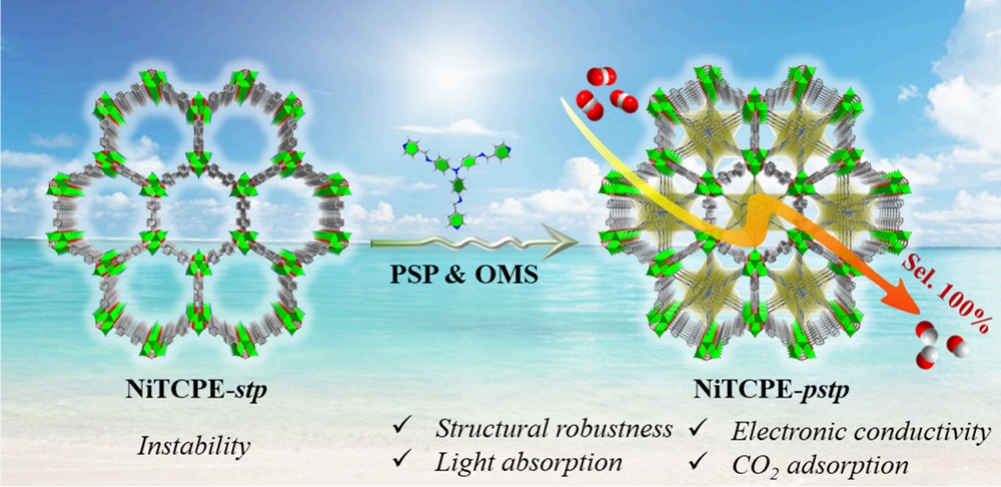

Rational design and regulation of atomically precise photocatalysts are essential for constructing efficient photocatalytic systems tunable at both the atomic and molecular levels. Herein, we propose a platform-based strategy capable of integrating both pore space partition (PSP) and open-metal sites (OMSs) as foundational features for constructing high-performance photocatalysts. We demonstrate the first structural prototype obtained from this strategy: pore-partitioned NiTCPE-*pstp* (TCPE = 1,1,2,2-tetra(4-carboxylphenyl)ethylene, *pstp* = partitioned *stp* topology). Nonpartitioned NiTCPE-*stp* is constructed from six-connected [Ni_3_(μ_3_–OH)(COO)_6_] trimer and TCPE linker to form 1D hexagonal channels with six coplanar OMSs directed at channel centers. After introducing triangular pore-partitioning ligands, half of the OMSs were retained, while the other half were used for PSP, leading to unprecedented microenvironment regulation of the pore structure. The resulting material integrates multiple advanced properties, including robustness, wider absorption range, enhanced electronic conductivity, and high CO_2_ adsorption, all of which are highly desirable for photocatalytic applications. Remarkably, NiTCPE-*pstp* exhibits excellent CO_2_ photoreduction activity with a high CO generation rate of 3353.6 μmol g^–1^ h^–1^ and nearly 100% selectivity. Theoretical and experimental studies show that the introduction of partitioning ligands not only optimizes the electronic structure to promote the separation and transfer of photogenerated carriers but also reduces the energy barrier for the formation of *COOH intermediates while promoting CO_2_ activation and CO desorption. This work is believed to be the first example to integrate PSP strategies and OMSs within metal–organic framework (MOF) photocatalysts, which provides new insight as well as new structural prototype for the design and performance optimization of MOF-based photocatalysts.

## Introduction

Conversion of CO_2_ into value-added products through chemical methods has attracted great attention to address energy and environmental issues.^1−5^ Particularly, photochemical CO_2_ conversion by imitating natural photosynthesis is a useful method to achieve carbon neutrality and sustainable development.^6−9^ However, due to inherent inertness of CO_2_ and multielectron transfer process, photocatalytic CO_2_ conversion is still in an urgent need for better catalysts.^10,11^ Although various photocatalysts have been studied in photocatalytic CO_2_ reduction, low activity, limited stability, competing side reactions, and less-well-defined catalytic sites and mechanisms have hindered practical applications.^12−14^ The development of photocatalytic systems with atomically precise structures and excellent overall performance remains challenging.

Metal–organic frameworks (MOFs), as atomically precise crystalline porous materials, can allow diverse structural design and customization through the assembly between predesigned organic ligands and inorganic units.^15−17^ In addition, the combination of high specific surface area and adjustable porosity with controllable photoelectric activity makes it a promising CO_2_ photoreduction platform.^18−23^ Although the vision of using MOFs materials as photocatalysts for CO_2_ green conversion is beautiful, there are formidable obstacles. To date, most MOF-based photocatalysts have poor stability in aqueous solutions due to relatively unstable coordination bonds.^24,25^ For large-pore MOFs, although the high porosity is conducive to CO_2_ mass transfer, the framework is more prone to collapse and may be less efficient for CO_2_ capture. Moreover, the relatively low charge separation efficiency has diminished photocatalytic performances.^26,27^ Therefore, seeking innovative methods to simultaneously enhance stability, optimize the pore structure, and promote charge separation is key for synthesizing efficient MOF photocatalysts. However, the design scheme, with the potential to revolutionize precise chemical synthesis and controllable material fabrication, not only appeals greatly to researchers in the fields of chemistry and materials but also poses significant challenges.

Previous studies have shown that the stability of MOFs can be enhanced by increasing connectivity and network rigidity.^28,29^ High-nuclearity metal clusters were used as multiconnected nodes to synthesize stable MOFs, such as UiO-66, PCN-222, MOF-525, but this method is less effective for 3d transition metals (e.g., Fe^3+^, Co^2+^, and Ni^2+^) that are often needed as catalytic sites.^30−32^ Another method to increase connectivity is to introduce a second ligand on OMSs. For example, Yang et al. constructed ultrastable Cr-MOFs by introducing 2,4,6-tri(4-pyridyl)-1,3,5-triazine (tpt) ligands to increase the connectivity based on the pore space partition (PSP) strategy.^33^ As a structure and pore-channel modification method, the PSP strategy can not only enhance rigidity and stability by introducing supporting ligands but also can enhance CO_2_ adsorption, which can be described as killing two birds with one stone.^34,35^ More importantly, the introduction of partitioning ligands can improve the microenvironment and photoelectric activity, which is expected to promote the separation and transfer of photogenerated charges to optimize catalytic properties and product selectivity. Consequently, the PSP-strategy-derived MOF materials can be used as a multifunctional platform to meet the multiple challenges faced by MOF-based photocatalytic materials.

**Scheme 1:**
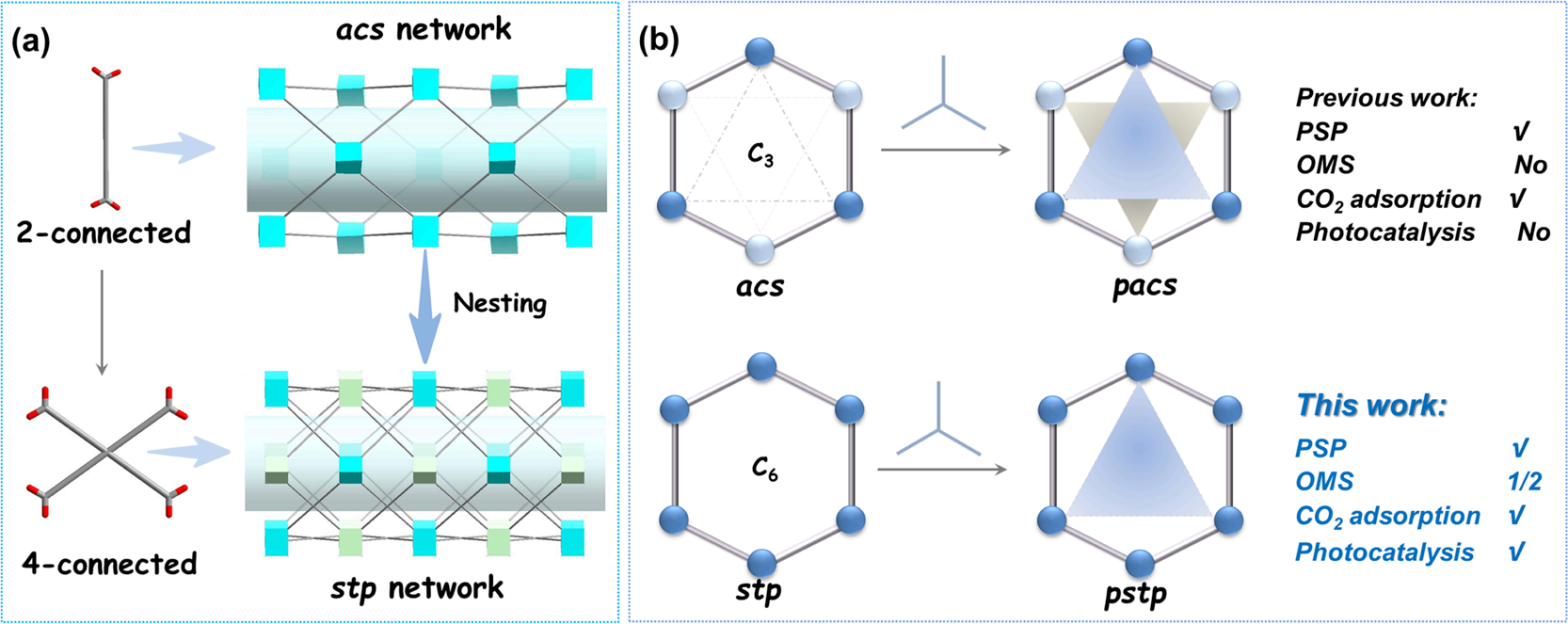
(a, b) Illustration of a New Design Strategy to Synthesize a Robust and Efficient *pstp*-MOF Photocatalyst Integrating Dual Features of PSP and OMSs

As a typical representative of PSP strategy, the *pacs* family has been widely used in gas adsorption and separation.^36−40^ The *pacs* materials are based on a MIL-88 structural prototype, an *acs* network connected by ditopic ligands such as dicarboxylates (Scheme 1a). In *pacs*, the triangular pore partition ligands inevitably result in the complete occupation of open-metal sites after their introduction, which can be unfavorable for catalytic reactions. This contradiction between the PSP and OMS presents a significant dilemma, posing considerable challenges and limitations for PSP-directed MOFs in the field of catalysis. Consequently, it is crucial to integrate the PSP and OMS into a MOF platform to establish a new family of PSP-MOFs capable of retaining catalytic sites with precisely defined structures and adjustable performance. For the MIL-88 *acs*-structure, only three trimer clusters are located on the *C*_3_ symmetrical plane to provide three sites in the 1D hexagonal channel. We hypothesized that if the two structures are nested based on the existing structure, the 1D channel will be formed but with six OMSs facing the center of the pore channel instead of three in the *acs* (Scheme 1b). We further hypothesized that the nested structure can be seen as a cross-arrangement of two dicarboxylates which can be substituted with a tetracarboxylic acid component. Therefore, by replacing two dicarboxylic acid ligands with a tetracarboxylic acid ligand, a novel structural prototype with *stp*-topology will be provided for executing the PSP strategy with more OMSs.

Based on the above hypotheses, in this work, we designed and selected TCPE as the main ligand to construct a MOF with *stp* topology, namely, NiTCPE-*stp*. As expected, NiTCPE-*stp* shows a honeycomblike hexagonal arrangement of six OMSs evenly distributed in the plane. In the next step, a pore-partition ligand, triangular tris(4-pyridin-4-ylmethylene)amino)-phenyl)amine (TPAPA), was chosen based on symmetry and size matching to successfully realize the integration of PSP and OMSs within NiTCPE-*pstp* MOF (*pstp* = partitioned *stp*). Remarkably, this strategy effectively combines the advantages of PSP and OMS, resulting in significant improvements in the structural robustness, gaseous sorption, and photoelectric activity of NiTCPE-*pstp*. Serving as the inaugural member guided by this approach, NiTCPE-*pstp* displays excellent photocatalytic activity (3353.6 μmol g^–1^ h^–1^) and ultrahigh selectivity (∼100%) and is among the most advanced MOF-based photocatalysts. Detailed photoelectric studies and density functional theory (DFT) calculations were performed to reveal the catalytic mechanism and structure–activity relationship as described below. This work represents an unprecedented example of integrating PSP and OMSs into MOF photocatalysts for multilevel optimization of the pore structure, microenvironment, and electronic structure to enhance catalytic performances. Based on this new atomically precise PSP strategy, we demonstrate that the structures and functions of MOFs can be adjusted, thus opening up new avenues for boosting performance and customizing applications.

## Results and Discussion

**Figure 1:**
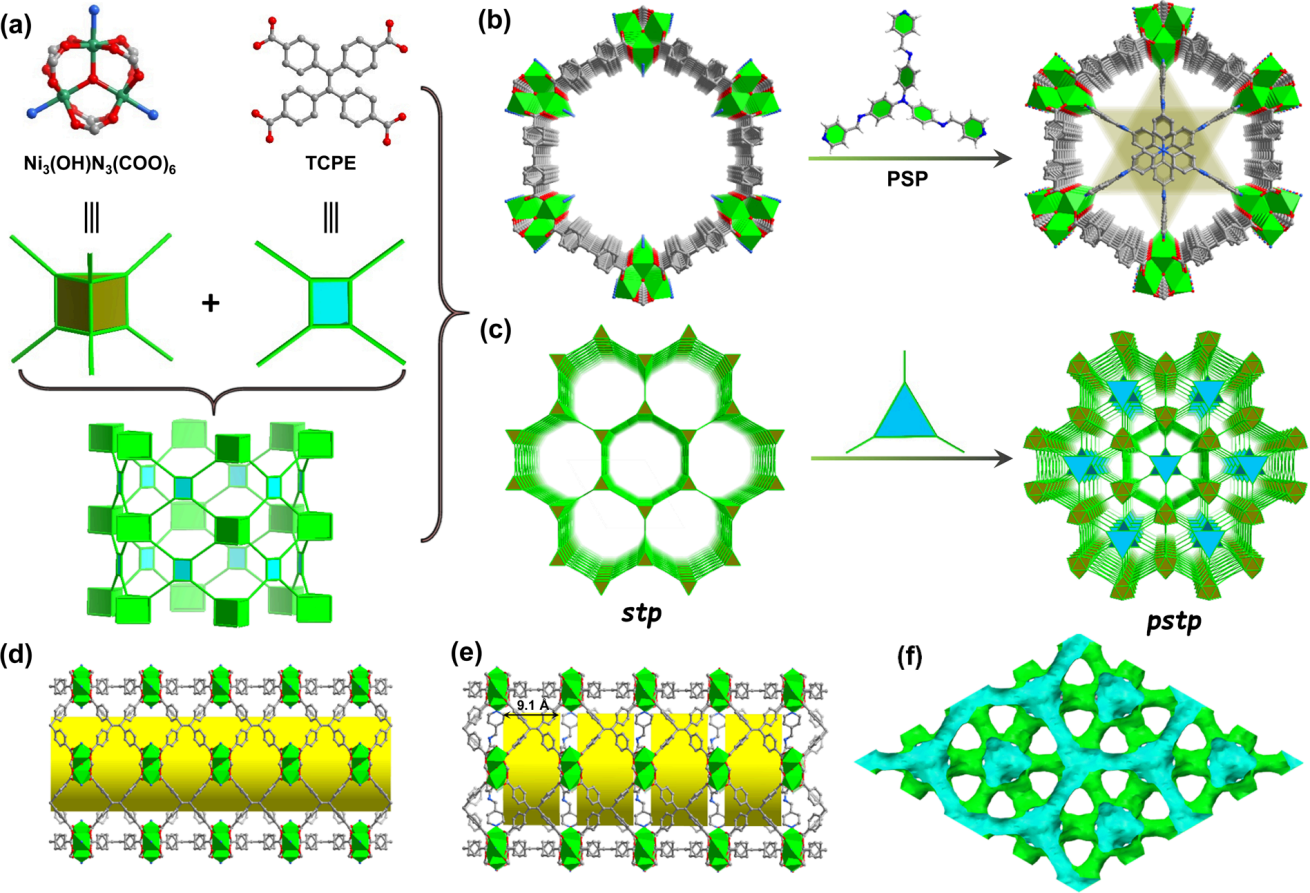
Illustration of the structural components and PSP in NiTCPE-*pstp*. (a) Assembly of NiTCPE-*stp* via Ni_3_ trimer building blocks with TCPE ligands and their simplification. (b, c) PSP through symmetry-matching regulated ligand insertion and their polyhedral drawing of the connected network viewed along the crystallographic *c*-axis. (d, e) Side view of the 1D cylindrical channel and corresponding segments before and after partition. (f) Pore surfaces of NiTCPE-*pstp* viewed along the *c*-axis.

Light green crystals of NiTCPE-*stp* were prepared by solvothermal reaction of Ni(NO_3_)_2_·6H_2_O, TCPE, and pyridine (Py) in a DMA/MeOH/H_2_O solution. When pyridine was replaced with pore-partitioning TPAPA ligands, a similar reaction led to dark-red crystals of NiTCPE-*pstp*. Single crystal X-ray diffraction analysis revealed that NiTCPE-*stp* and NiTCPE-*pstp* crystallize in the trigonal *P*–3 and hexagonal *P*622 space groups, respectively (Table S1). As shown in Figure 1a, the 3D network of NiTCPE-*stp* is assembled by TCPE ligands and trinuclear clusters of [Ni_3_(μ_3_–OH)(COO)_6_(Py)_3_]. In the Ni_3_ trimer, each Ni atom is six-coordinated by five oxygen atoms and one nitrogen atom in an octahedral environment with Ni–O lengths in the range of 1.994(3)–2.051(3) Å. Interestingly, each Ni_3_ trimer is connected by TCPE ligands to generate a 3D framework with honeycomb 1D nanosized channels and rhombic-like window along *c*-axis and *a*-axis direction with the size of ca. 15.97 × 15.97 Å^2^ and 13.0 × 10.1 Å^2^, respectively (Figures 1b–d, S1–S2). Considering TCPE ligands and Ni_3_ trimers as 4- and 6-connected nodes, respectively, the network can be reduced to a 4,6-connected *stp* topology with the symbol {4^4.6^2}_3_{4^9.6^6}_2_ (Figure 1c). It is worth noting that the hexagonal channels are similar to those in MIL-88 structures but consist of six trinuclear clusters in the same plane, which provides an opportunity to achieve PSP while preserving more OMSs. Therefore, TPAPA was designed and screened as partitioning ligands based on the size requirement and symmetry matching to implement the PSP strategy (Figure S3). As expected, structural analysis of NiTCPE-*pstp* shows that three Ni sites in the hexagonal plane are coordinated with three pyridine groups of one TPAPA ligand to complete the pore partition, while the other three Ni sites are coordinated with H_2_O molecules, which can be easily removed to form the OMSs (Figure S4). It is noteworthy that by introducing partitioning ligands, half of the metal sites are retained, while the other metal sites are used for PSP, thereby achieving the regulation of the pore structure and microenvironment, in a way different from the previously reported *pacs* types. Fourier transform infrared (FT-IR) spectrum of NiTCPE-*pstp* shows two typical characteristic peaks at 1623 and 1322 cm^–1^ assigned to C = N and C–N bonds, respectively, confirming the implantation of TPAPA into the pores as a partitioning ligand (Figure S5). To our knowledge, embedding the *C*_3_-symmetric partitioning ligands in the *C*_6_ plane to achieve the synchronous optimization of the PSP strategy and the OMSs is unprecedented. Compared with NiTCPE-*stp*, the original hexagonal 1D channels were evenly segmented into continuous segments after the introduction of TPAPA, resulting in numerous cage-like pore cavities with the size of ca. 21.2 × 21.2 × 9.1 Å^3^, which shows the potential for improved channel microenvironment and enhanced confinement effect (Figures 1e and S6). Without considering solvent molecules, the free volume (*V*_void_) of NiTCPE-*pstp* was calculated to be 58.5% by PLATON, slightly lower than that of NiTCPE-*stp* (ca.78.7%) but still larger than that of many reported highly microporous MOFs. The pore surface of NiTCPE-*pstp* viewed along the *c*-axis is shown in Figure 1f, and the interpenetrated pore structure is conducive to efficient mass transfer in catalytic process.

X-ray photoelectron spectroscopy (XPS) studies of NiTCPE-*stp* and NiTCPE-*pstp* were performed to investigate the valence states and coordination environments of the metal ions (Figure S7). The XPS analysis shows that the peaks at 856.13 and 855.93 eV are assigned to the Ni(II) 2p_3/2_ orbitals of NiTCPE-*stp* and NiTCPE-*pstp*, respectively, indicating both MOFs have anionic frameworks. The ^1^H NMR spectroscopy further proved the existence of counterions [NH_2_(CH_3_)_2_]^+^ in these MOFs (Figure S8). Importantly, compared with the coordination-saturated NiTCPE-*stp*, the Ni 2p_2/3_ peak of NiTCPE-*pstp* is shifted by 0.2 eV toward the high-energy direction, which indicates the existence of open active sites unoccupied by Py groups after PSP.^41^ Powder X-ray diffraction (PXRD) results show that the diffraction patterns of NiTCPE-*stp* and NiTCPE-*pstp* were well matched with the simulated patterns, supporting their phase purity (Figure S9). Thermogravimetric analysis results show that these compounds maintained good thermal stability before and after pore partitioning (Figure S10).

**Figure 2:**
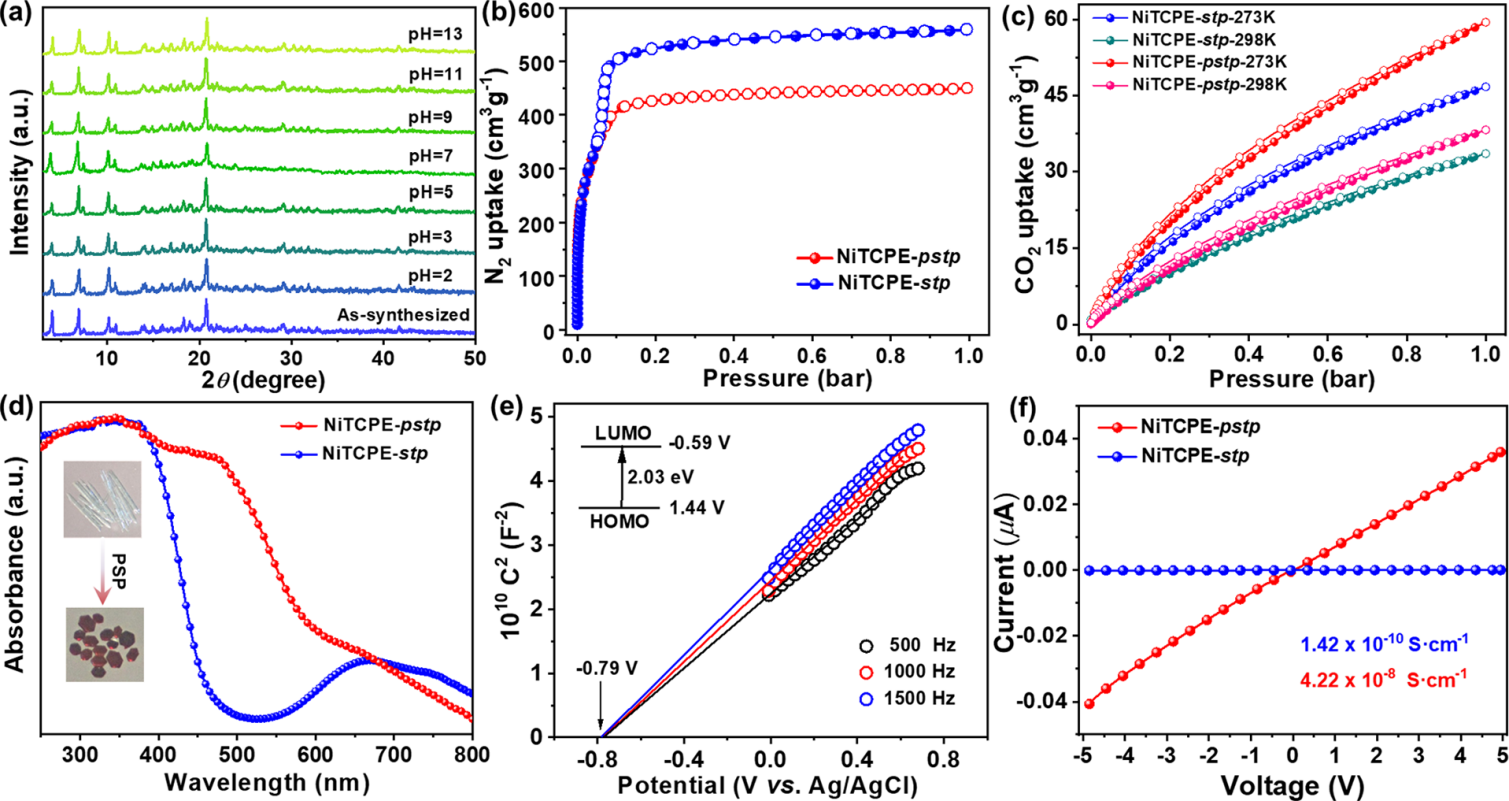
(a) PXRD patterns of NiTCPE-*pstp* after immersion in aqueous solution at pH from 2 to 13. (b, c) N_2_ adsorption isotherms (at 77 K) and CO_2_ adsorption isotherms (at 273 and 298 K) of NiTCPE-*pstp* and NiTCPE-*stp*. (d) UV–vis absorption spectra of NiTCPE-*pstp* and NiTCPE-*stp*; inset: photographs of single crystals before and after PSP. (e) Mott–Schottky plot of NiTCPE-*pstp*. (f) *I*–*V* curves of NiTCPE-*pstp* and NiTCPE-*stp* at room temperature.

To further evaluate the effect of PSP for chemical stability, we tested the PXRD patterns of NiTCPE-*pstp* and NiTCPE-*stp* after immersion in water or organic solvents. The results show that NiTCPE-*stp* has a high stability in organic solvents but quickly loses its crystal state in water (Figure S11). In contrast, NiTCPE-*pstp* shows high framework robustness, which not only can survive in water and organic solvents for 1 week but also keep good stability and crystal state in aqueous solutions with a wide pH range (pH = 2–13) (Figures 2a and S12). Considering the similar cluster node of Ni_3_ trimer and the same organic linker of TCPE, the enhanced chemical stability of NiTCPE-*pstp* could be attributed to the support from multiple binding of pore space-partitioning ligand, the high connectivity, and the network rigidity.^42^ The porosity of MOF materials before and after using the PSP strategy was studied by nitrogen adsorption at 77 K. Both NiTCPE-*pstp* and NiTCPE-*stp* show typical type-I adsorption isotherms, indicating their microporous characteristics (Figure 2b). It is worth noting that compared with the unpartitioned NiTCPE-*stp*, the adsorption capacity of NiTCPE-*pstp* was reduced from 560 to 450 cm^3^ g^–1^, further suggesting the successful implantation of partitioning ligands. The Brunauer–Emmett–Teller (BET) surface areas of NiTCPE-*stp* and NiTCPE-*pstp* are calculated to be 1610 and 1347 m^2^ g^–1^, respectively. In addition, the CO_2_ uptakes were investigated at 273 and 298 K to evaluate the optimization of its adsorption by the PSP strategy. As shown in Figure 2c, the CO_2_ uptakes values of NiTCPE-*pstp* were 59.5 cm^3^ g^–1^ at 273 K and 38.2 cm^3^ g^–1^ at 298 K, which were higher than that of NiTCPE-*stp* (46.7 cm^3^ g^–1^ at 273 K and 33.5 cm^3^ g^–1^ at 298 K), demonstrating the enhanced CO_2_ affinity after PSP. Correspondingly, the isosteric heat of adsorption (*Q*_st_) values were 28.11 and 21.57 kJ mol^–1^ for NiTCPE-*pstp* and NiTCPE-*stp* based on single-component isotherms at 273 and 298 K, respectively (Figure S13). The higher *Q*_st_ of NiTCPE-*pstp* shows the stronger framework–gas interaction, which is conducive to the fixation and activation for CO_2_.^43^

The photoelectric properties of NiTCPE-*pstp* and NiTCPE-*stp* were studied to further reveal the multilevel regulation for physical and chemical properties by the PSP strategy. As shown in Figure 2d, the solid-state UV–vis absorption spectrum of NiTCPE-*stp* shows an obvious visible light absorption in the range 400–800 nm. In contrast, the visible-light-harvesting ability of NiTCPE-*pstp* was greatly enhanced with the absorption edge up to 610 nm, which is derived from the introduction of partitioning ligand and their functional optimization and is ideal for photocatalytic reactions (Figure S14). As observed, the crystal color changed from the original light green to dark red after PSP, which is consistent with its absorption spectrum. The band gap energy (*E*_g_) of NiTCPE-*pstp* is obtained as 2.03 eV according to the Tauc plot, which is far narrower than that of NiTCPE-*stp* (2.82 eV) (Figure S15). These results show that the implementation of the PSP strategy can not only adjust the shape and size of pore space but also optimize the energy band structure and electronic configuration. To further understand the electronic structural characteristics of NiTCPE-*pstp* and NiTCPE-*stp*, Mott–Schottky measurements were conducted at 500, 1000, and 1500 Hz frequencies. As shown in Figures 2e and S16, the Mott–Schottky curve shows the typical n-type semiconductor features of these MOF materials, and the flat potentials were evaluated as −0.79 and −0.95 V vs Ag/AgCl for NiTCPE-*pstp* and NiTCPE-*stp*, respectively. Since the position of conduction band minimum (CBM) is close to the flat band potential for *n*-type semiconductor, the CBM positions of NiTCPE-*pstp* and NiTCPE-*stp* were determined to be −0.59 and −0.75 V vs NHE.^44,45^ Accordingly, their valence band maximum (VBM) positions are calculated to be 1.44 and 2.07 V vs NHE based on the band gap energy, respectively. It is worth noting that their CBM positions were more negative than the reduction potential of CO_2_ to CO (−0.53 V vs NHE, pH = 7), showing the thermodynamic feasibility for photocatalytic CO_2_ reduction. In addition, the *I*–*V* curves of NiTCPE-*pstp* and NiTCPE-*stp* were measured at room temperature (RT) to reveal their solid-state electric conductivity. As shown in Figure 2f, the electric conductivity of NiTCPE-*stp* was determined to be 1.42 × 10^–10^ S cm^–1^ at RT. In contrast, NiTCPE-*pstp* showed a significantly enhanced conductivity of 4.22 × 10^–8^ S cm^–1^, which increased nearly 300 times compared to that of NiTCPE-*stp*. Enhanced electrical conductivity can greatly promote the charge transfer for the photocatalytic process.

**Figure 3:**
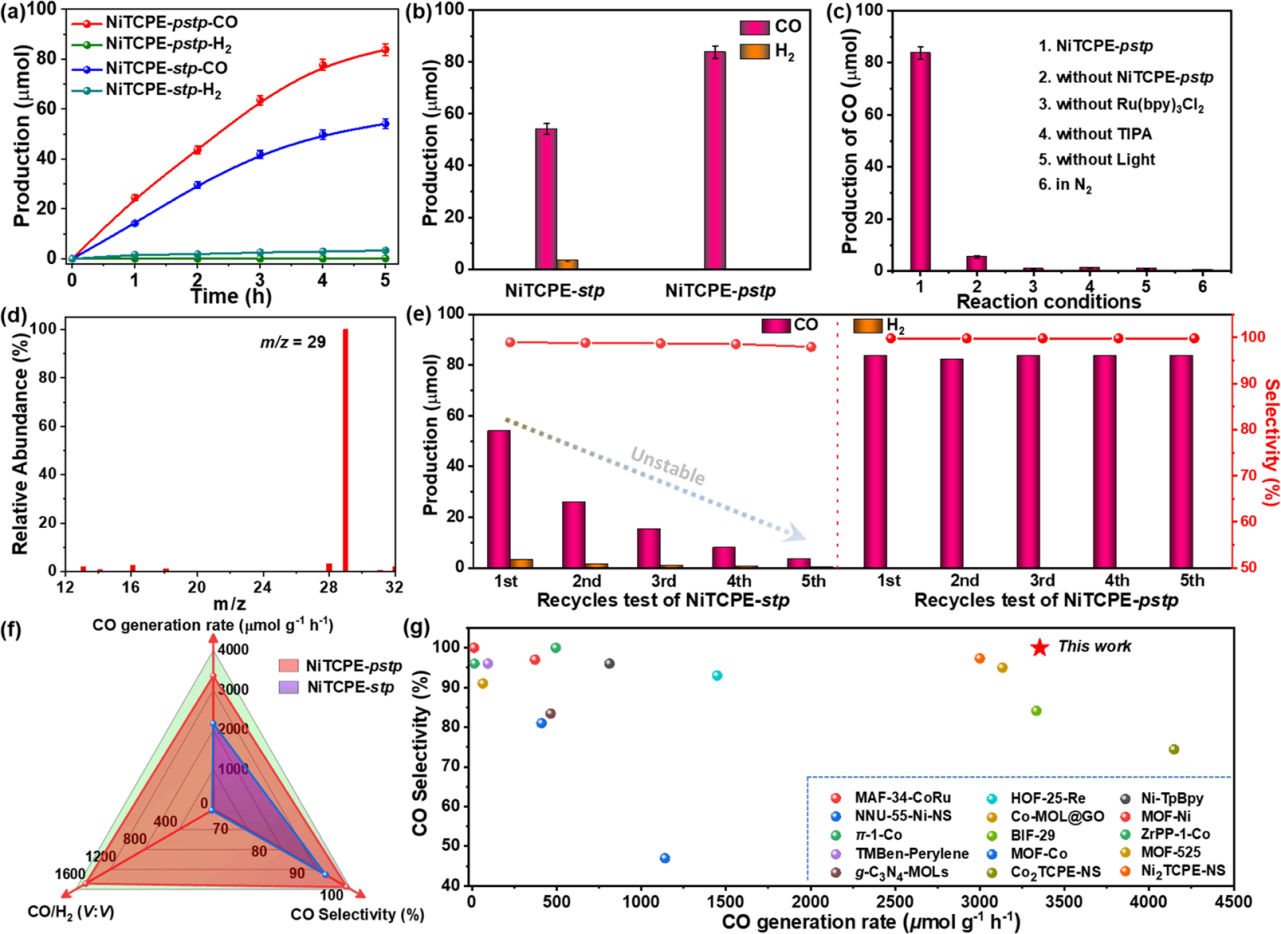
Photocatalytic CO_2_ reduction performances of NiTCPE-*pstp* and NiTCPE-*stp*. (a) Time-dependent photocatalytic CO and H_2_ evolution. (b) Comparison of the amount of CO and H_2_ production within 5 h. (c) CO yields of NiTCPE-*pstp* under different conditions. (d) Mass spectrometry analysis of ^**13**^CO from ^**13**^CO_2_ isotope tracer experiment. (e) Recycles test for CO_2_ photoreduction. (f) Radar map of photocatalytic comprehensive performances comparison. (g) Comparison of the performances with other MOFs-based crystalline photocatalysts for CO_2_ reduction to CO.

Based on the above results, the atomically precise PSP strategy integrates a variety of advanced advantages, including robustness, wider absorption range, enhanced conductivity, matching potential, and high CO_2_ adsorption, which prompted us to explore the photocatalytic CO_2_ reduction performance of NiTCPE-*pstp*. The photocatalytic CO_2_ reduction experiments were carried out in a CH_3_CN/H_2_O mixed solvent system with [Ru(bpy)_3_]Cl_2_·6H_2_O (bpy = 2,2′-bipyridine) as the photosensitizer and triisopropanolamine (TIPA) as an electron donor under the visible-light radiation (λ ≥ 420 nm). As shown in Figure 3a, NiTCPE-*stp* exhibits significant photocatalytic CO_2_ reduction activity to generate CO accompanied by a small amount of H_2_ byproduct. The yields of reduction products gradually increase over time to achieve stability with a maximum amount of 54.04 μmol for CO and 3.19 μmol for H_2_ at 5 h. The average generation rates of CO and H_2_ of NiTCPE-*stp* were observed to be 2161.5 and 127.6 μmol g^–1^ h^–1^ after 5 h of irradiation, respectively. In contrast, NiTCPE-*pstp* shows a higher CO yield of 83.84 μmol and almost negligible yield of H_2_ (Figure 3b). The average CO generation rate of NiTCPE-*pstp* was determined to be 3353.6 μmol g^–1^ h^–1^, which is 1.5 times than that of NiTCPE-*stp*. More importantly, NiTCPE-*pstp* exhibits approximately 100% (99.95%) CO selectivity with ultrahigh CO/H_2_ ratio of 1625, which exceeds that of NiTCPE-*stp* (selectivity of 94.4%, CO/H_2_ ratio of 17). Under identical conditions, the TPAPA ligand shows diminished photocatalytic CO_2_ reduction activity, producing a maximum quantity of 5.94 μmol for CO, which is significantly lower than that of NiTCPE-*pstp*, indicating that the enhanced photocatalytic activity of NiTCPE-*pstp* comes from multilevel optimization of the pore structure, microenvironment, and electronic structure after PSP, rather than a mere combination of pore-partitioning TPAPA ligand and NiTCPE-*stp* (Figure S17). The ^1^H NMR of the solution after the catalytic reaction was tested to detect any possible liquid phase product, and the results showed that no formic acid or alcohol species were observed, suggesting that CO is the only carbon product of photocatalytic CO_2_ reduction (Figure S18).

Additionally, a series of comparative experiments under different conditions was systematically tested to reveal the indispensable role of each component in the photocatalytic process. As shown in Figures 3c and S19, the reaction is difficult to carry out with no NiTCPE-*pstp* in the system, indicating the main role of the catalyst for photocatalytic CO_2_ reduction. In the absence of TIPA, the reaction almost stopped, indicating that the electron sacrificial agent is necessary for the catalytic reaction. Similarly, the catalyst has almost no reactivity without light or [Ru(bpy)_3_]Cl_2_, which shows that the reaction of CO_2_ to CO is a photocatalytic reaction and requires a sensitization process. The above results are consistent with most of the previous studies on MOF-based photocatalytic materials.^46,47^ In addition, the catalytic activity in a N_2_ atmosphere was also investigated, and the results showed that no CO products were detected revealing the generated carbon product came from CO_2_ rather than the decomposition of the catalyst or solvent. Furthermore, the ^13^CO isotope labeling experiment was tested to further prove the source of the CO product by replacing CO_2_ with ^13^C-enriched CO_2_, and the product CO was analyzed by gas chromatography–mass spectrometry (GC–MS). As a result, the main peak detected with the *m*/*z* of 29 corresponds to ^13^CO, which exhibits that CO_2_ is the only source of carbon for the reduction product without pollution from other organic components (Figure 3d).

Recycle stability is also an important index for the evaluation of photocatalytic performance in addition to the catalytic rate and product selectivity. To investigate the stability of NiTCPE-*stp* and NiTCPE-*pstp*, we conducted the cyclic experiments five times under the same conditions. As shown in Figures 3e and S20, the CO yields of NiTCPE-*stp* gradually decreased with the increase of cycle number, while it maintained a relatively stable selectivity. After five cycles, the CO yield only became less than one-fifth of the original, which indicates that NiTCPE-*stp* gradually lost activity during the catalysis process. In particular, the changed PXRD pattern and insignificantly low N_2_ adsorption of NiTCPE-*stp* after catalysis further illustrate its limited structural and performance stability (Figures S21–S22). Different from NiTCPE-*stp*, the recycles experiment results of NiTCPE-*pstp* show that the CO yields have almost no attenuation with the cycle number and still maintain the high CO yield of 83.74 μmol and ∼100% selectivity after five cycles, demonstrating the long-term and stable photocatalytic activity (Figures 3d and S23). The excellent cyclic stability that is proven by the unchanged PXRD patterns before and after photocatalysis could come from the higher structural stability caused by the introduction of partitioning ligands and the enhanced network rigidity (Figure S24). Moreover, FT-IR and XPS spectroscopy illustrate the consistency of valence state and coordination environment of Ni^2+^ in NiTCPE-*pstp* before and after photocatalysis (Figures S25 and S26). Correspondingly, the scanning electron microscopy and high-resolution transmission electron microscopy images before and after photocatalysis further reveal that the morphology is well maintained without any nanoparticles observed, suggesting the photocatalytic activity from NiTCPE-*pstp* rather than from the oxide or metal nanoparticles produced by its decomposition (Figures S27 and S28). These results further confirm the excellent structural and performance stability of NiTCPE-*pstp* and its potential and feasibility for long-lasting and efficient photocatalysis. To provide a visual contrast showing advantages of PSP, the comparison of comprehensive photocatalytic performances for NiTCPE-*pstp* and NiTCPE-*stp* is displayed in Figure 3f. These results indicate that the introduction of PSP ligands not only enhances the overall performance, including superior CO generation rate, ultrahigh CO/H_2_ ratio, and nearly 100% selectivity but also greatly improves the structural stability and performance stability for the photocatalyst, which is unprecedented. Furthermore, such remarkable photocatalytic performance of NiTCPE-*pstp* is among the best MOF-based crystalline materials for the reduction of CO_2_ to CO, which provides a new perspective and strategy to design and modify crystalline MOFs catalysts at atomic and molecular levels (Figure 3g, Table S4).

**Figure 4:**
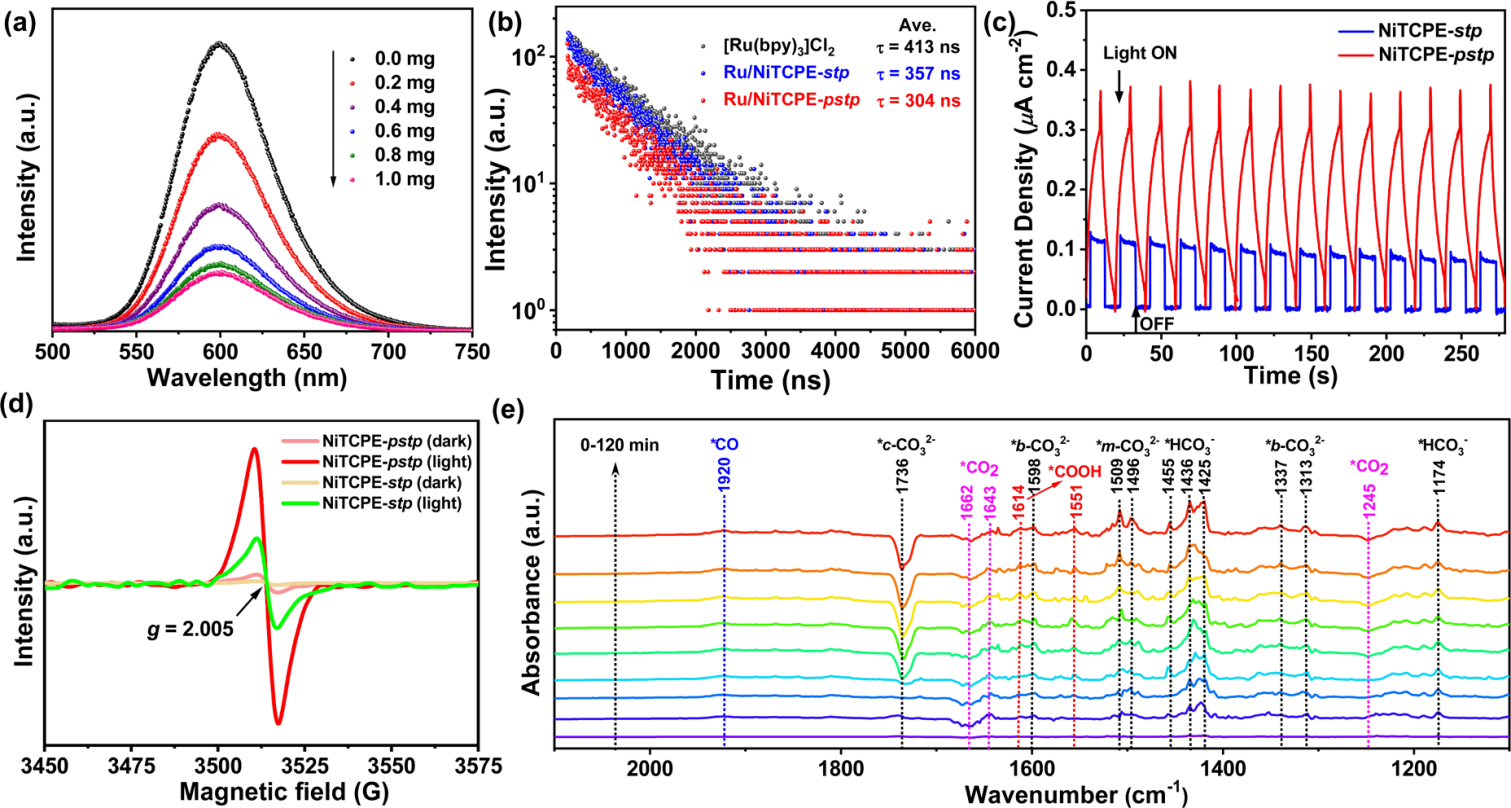
(a) Steady-state PL emission spectrum of [Ru(bpy)_3_]Cl_2_ with the addition of NiTCPE-*pstp*. (b) Time-resolved PL decay spectra of [Ru(bpy)_3_]Cl_2_ with or without NiTCPE-*pstp* and NiTCPE-*stp*. (c) Transient photocurrent responses of NiTCPE-*pstp* and NiTCPE-*stp*. (d) In situ EPR spectra of NiTCPE-*pstp* and NiTCPE-*stp* in dark and light. (e) In situ DRIFTS spectra of NiTCPE-*pstp* in the atmosphere of CO_2_ and H_2_O vapor under visible-light irradiation.

To gain deeper insight into the separation of photogenic charge before and after PSP, the photoluminescent (PL) quenching experiments were carried out in the CH_3_CN/H_2_O mixed system containing [Ru(bpy)_3_]Cl_2_ with the photocatalyst or TIPA. As shown in Figures 4a and S29, the steady-state PL emission intensity gradually decreases with the addition of NiTCPE-*pstp*, which indicates that photogenerated electrons can be transferred from the excited state of [Ru(bpy)_3_]Cl_2_ to NiTCPE-*pstp*. In contrast, the PL quenching effect of NiTCPE-*stp* is much weaker than that of NiTCPE-*pstp*, suggesting the enhanced charge transfer ability after PSP. Furthermore, no significant PL quenching was found with increasing the amount of TIPA, further exhibits that the excited state photosensitizer can be oxidized and quenched by catalyst to realize the photogenerated electrons transfer (Figure S30).^48^ Time-resolved PL decay spectra reveal that the average lifetime of [Ru(bpy)_3_]Cl_2_ is calculated as 413 ns, which is significantly longer than the lifetimes after the addition of NiTCPE-*pstp* and NiTCPE-*stp*, fitted to be 304 and 357 ns, respectively (Figure 4b). The shorter PL lifetime illustrates the photogenerated electrons can be transferred quickly, further indicating the stronger ability of NiTCPE-*pstp* for inhibiting the recombination of photogenerated carriers, thereby showing higher photocatalytic activity.^49^ Transient photocurrent responses were tested to further explore the separation efficiency of photogenerated electrons and holes, and the results showed that NiTCPE-*pstp* gives a good photoelectric response with the current density of 0.3 μA cm^–2^, which is close to three times that of NiTCPE-*stp*, suggesting the significantly improved photogenerated charge separation after PSP (Figure 4c). Moreover, electrochemical impedance spectroscopy measurements were performed to evaluate the internal resistance of the charge transfer process. As shown in the Nyquist curves (Figure S31), NiTCPE-*pstp* displays a smaller semicircular diameter than NiTCPE-*stp*, signifying a lower charge transfer resistance in NiTCPE-*pstp*, which is favorable for photogenerated electron transfer as well as photogenerated charge separation. The above findings further prove that the PSP strategy can effectively promote the separation of photogenic electrons and holes and improve the migration rate of the photogenic carrier to obtain efficient photocatalytic CO_2_ reduction activity.

To further unveil the charge transfer behavior and surface-active species during the photocatalytic process over NiTCPE-*pstp* and NiTCPE-*stp*, in situ electron paramagnetic resonance (EPR) measurements were performed with and without light irradiation. As shown in Figure 4d, the EPR spectrum of NiTCPE-*stp* shows a significant peak at *g* = 2.005 under visible light in contrast to the very weak signals detected in dark condition, belonging to paramagnetic Ni(I) species, which proves the proposed transfer pathway of the photogenic charge from the photosensitizer to [Ni_3_] clusters.^50,51^ Compared with NiTCPE-*stp*, the EPR spectrum of NiTCPE-*pstp* exhibits a significantly enhanced signal under visible light, three times that of NiTCPE-*stp*, which indicates the PSP strategy greatly promotes the generation and transfer of photogenic carriers, resulting in improved photocatalytic CO_2_ reduction activity. In situ diffuse reflectance infrared Fourier transform spectroscopy (DRIFTS) of NiTCPE-*pstp* was employed to detect the reactions intermediates of photocatalytic CO_2_ to CO. In the absence of light, we observed the occurrence of typical CO_2_ adsorbed intermediates on NiTCPE-*pstp* as depicted in Figure S32. Specifically, the peaks at 1174 and 1455 cm^–1^ can be attributed to *HCO_3_, while additional peaks indicate the presence of carbonate groups, including chelating bridged carbonate (*c*-CO_3_^2–^ 1736 cm^–1^), bidentate carbonate (*b*-CO_3_^2–^, 1357 and 1542 cm^–1^), and monodentate carbonate (*m*-CO_3_^2–^, 1503, 1560, and 1585 cm^–1^), resulting from the dissolution of CO_2_ in H_2_O.^52,53^ In addition, we identified the peaks at 1245 and 1639 cm^–1^ as active *CO_2_ intermediates. Over time, there was a noticeable enrichment of *c*-CO_3_^2–^ (1736 cm^–1^) and *CO_2_ (1245 cm^–1^) intermediates, indicating a strong interaction between CO_2_ and the NiTCPE-*pstp*.^54^ When the catalyst was irradiated with visible light, we observed the gradual appearance of intensified peaks, as shown in Figure 4e. The peak at 1736 cm^–1^ initially strengthened and then gradually declined, indicating the generation of bicarbonate or other species that likely serve as important intermediates in the photocatalytic CO_2_ reduction process.^55^ Notably, the peak at 1245 cm^–1^ gradually decreased with the progression of the reaction, suggesting the consumption of *CO_2_ to yield new intermediates. Moveover, we observed the gradual increase in the new peaks at 1551 and 1614 cm^–1^, which can be attributed to the *COOH, a key intermediate in the photoreduction of CO_2_ to CO, formed through the interaction between adsorbed *CO_2_ and protons.^56,57^ Under the influence of protons, the *COOH intermediate can further convert to *CO, as represented by the peak at 1920 cm^–1^, and ultimately leave the active site and develop into CO.^58^

**Figure 5:**
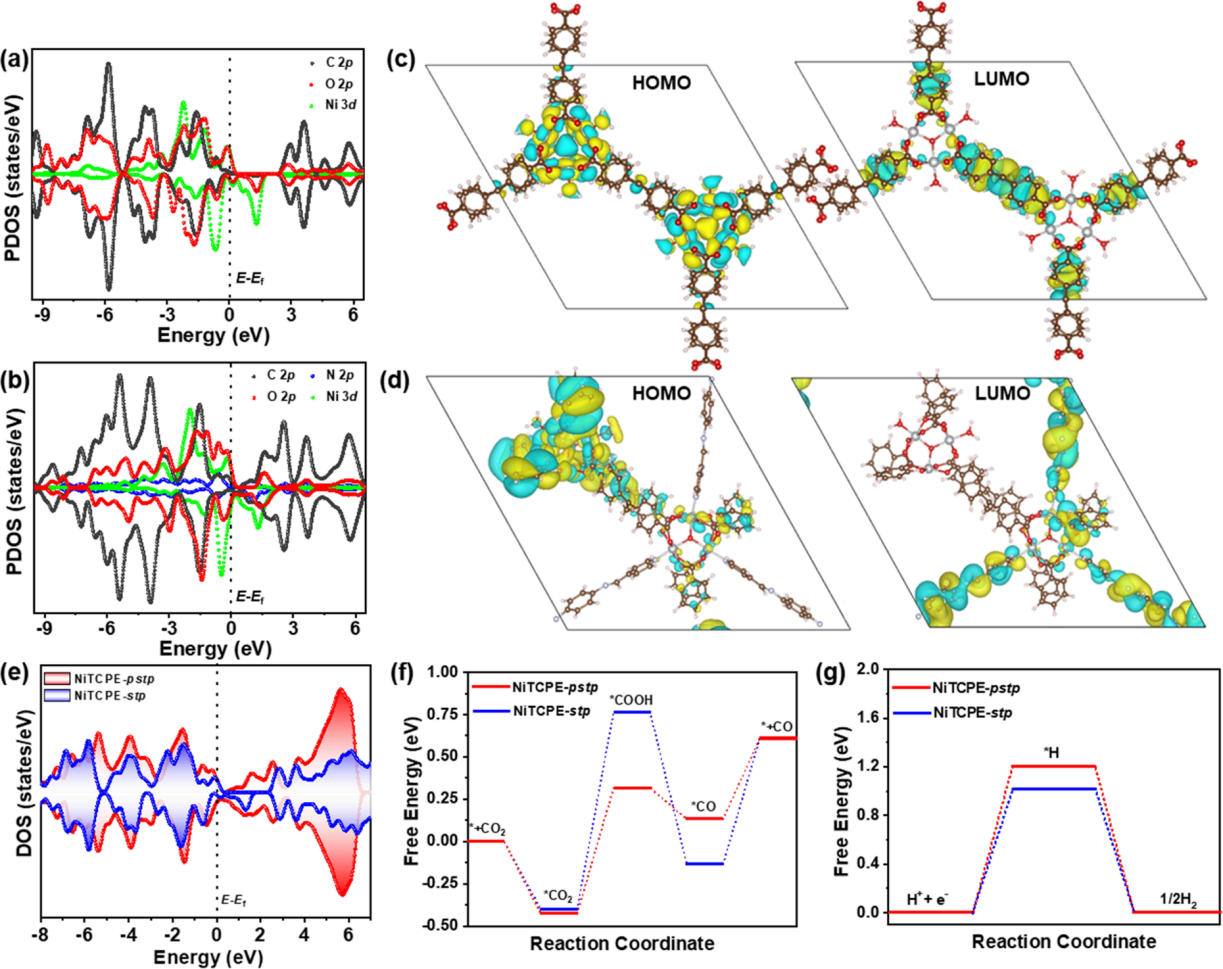
(a, b) PDOS of NiTCPE-*stp* and NiTCPE-*pstp*. (c, d) Molecular orbitals of HOMO and LUMO for NiTCPE-*stp* and NiTCPE-*pstp*. (e) DOS of NiTCPE-*stp* and NiTCPE-*pstp*. (f, g) Calculated free-energy of photocatalytic CO_2_ reduction to CO and H_2_ production for NiTCPE-*stp* and NiTCPE-*pstp*.

To further understand photocatalytic CO_2_ reduction over NiTCPE-*pstp*, DFT calculations were performed to illustrate the regulation of electronic structure and performance by the PSP strategy in achieving excellent photocatalytic activity and high selectivity. For the partial density of states (PDOS) of NiTCPE-*stp*, the VBM is mainly composed by the local electronic states of the [Ni_3_] trimer cluster, including the Ni-3d and O-2p states, while the CBM is mainly contributed by the C-2p and O-2p states from TCPE ligands, which matched with the spatial distribution of its highest occupied molecular orbital (HOMO) and lowest unoccupied molecular orbital (LUMO) (Figure 5a,c). Interestingly, significant changes in electronic structure were observed in NiTCPE-*pstp* when the PSP was implemented. Specifically, the PDOS of NiTCPE-*pstp* shows the composition of VBM is similar to that of NiTCPE-*stp*, but the CBM is contributed by N-2p and C-2p from the TPAPA ligands and significantly differs from the nonpartitioned structure (Figure 5b). Based on the orbital distribution of NiTCPE-*pstp*, it is easy to find that HOMO and LUMO are positioned on the [Ni_3_] cluster and the partitioning ligands of TPAPA, respectively (Figure 5d). Compared with NiTCPE-*stp*, the optimized orbital distribution after PSP is conducive to promoting the separation and transfer of photogenerated electrons and holes.^59^ More importantly, the introduction of TPAPA effectively increases the new states near the Fermi level and promotes the movement of CBM toward the Fermi level, indicating enhanced photocatalytic activity for NiTCPE-*pstp* compared to NiTCPE-*stp* (Figure 5e).^60^

In addition, theoretical calculations were also used to study the energy changes of the CO_2_ reduction to gain insight into the CO_2_ reduction process and mechanism. As shown in Figure S33, the adsorption energy of CO_2_ on NiTCPE-*pstp* is stronger than that of NiTCPE-*stp*, indicating the stronger adsorption affinity of NiTCPE-*pstp* for CO_2_, which is consistent with the enhanced CO_2_ uptakes values. As is widely known, photocatalytic CO_2_ reduction to CO entails several key steps. Initially, CO_2_ is adsorbed onto the catalyst of the MOF, forming an activated *CO_2_ intermediate. This intermediate then combines with H^+^ and e^–^ to generate the *COOH intermediate. Subsequently, the *COOH intermediate undergoes protonation, producing the *CO species, which is ultimately desorbed from the MOF, leading to the release of the CO product. It is worth noting that the formation of *COOH was highly endergonic processes, which is also the rate-determining step for reduction of CO_2_ to CO.^61^ Based on the calculated reaction free energies shown in Figures 5f and S34–S37, the NiTCPE-*pstp* exhibits a lower energy barrier for *COOH (0.74 eV) compared to the unpartitioned NiTCPE-*stp* (1.162 eV). This suggests that the introduction of TPAPA can effectively reduce the energy barrier for the *COOH intermediate and enhance its bonding ability with metal sites, thereby promoting the efficient photocatalytic CO_2_ reduction activity. Furthermore, the energy barrier for the conversion from *COOH to *CO in NiTCPE-*pstp* was determined to be −0.183 eV, indicating that this step occurs spontaneously. Additionally, NiTCPE-*pstp* demonstrates a lower energy barrier of 0.476 eV for the transition from *CO to CO compared to NiTCPE-*stp* (0.742 eV), highlighting the beneficial effect of PSP in promoting the desorption of CO from the active sites and enhancing the CO generation rate. Moreover, the free energy of the adsorbed H* intermediate on NiTCPE-*pstp* exhibits a higher energy barrier of 1.21 eV when compared with NiTCPE-*stp* (1.02 eV), indicating the efficient suppression of the hydrogen evolution side reaction following the introduction of PSP (Figure 5g).^62−64^ These results show that the implementation of the PSP strategy and the introduction of TPAPA can effectively reduce the energy barrier of CO_2_ reduction and inhibit photocatalytic hydrogen evolution, resulting in enhanced photocatalytic CO_2_ reduction activity and nearly 100% CO selectivity.

**Figure 6:**
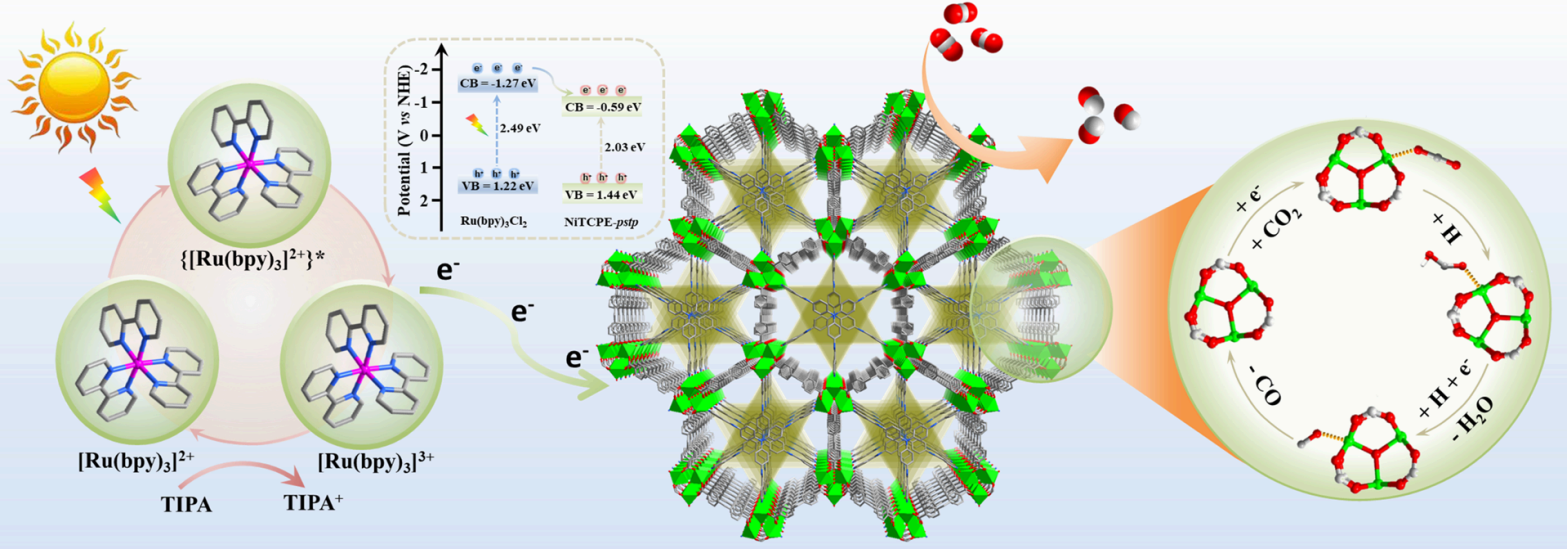
Charge transfer process of the photocatalytic system and the proposed mechanism of photocatalytic reduction of CO_2_ to CO over NiTCPE-*pstp*.

The proposed charge transfer process and photocatalytic mechanism have been elucidated through the combination of in situ EPR, in situ DRIFTS, and theoretical calculations, as shown in Figure 6. Upon excitation with visible light, the photosensitizer [Ru(bpy)_3_]^2+^ absorbs photons, leading to the formation of the [Ru(bpy)_3_]^2+^* species. It has been observed that the LUMO of NiTCPE-*pstp* is lower than that of [Ru(bpy)_3_]^2+^. Consequently, the LUMO electrons of the excited [Ru(bpy)_3_]^2+^* can be transferred to the surface of NiTCPE-*pstp*, supporting the findings from in situ monitored EPR. The transferred electrons are subsequently accepted by the CO_2_ molecules adsorbed at the open-metal site, resulting in the formation of *CO_2_ species. Subsequently, the *CO_2_ species are coupled to protons to generate the intermediate *COOH, which represents a crucial step in the photocatalytic reaction. The *COOH intermediate is further reduced to *CO with the concurrent participation of protons and electrons while removing a H_2_O molecule. Finally, *CO is desorbed at the metal site, releasing CO. Simultaneously, [Ru(bpy)_3_]^3+^ was generated from excited [Ru(bpy)_3_]^2+^* after transferring the electrons to NiTCPE-*pstp*, which is quickly quenched by the sacrificial agent to generate [Ru(bpy)_3_]^2+^ to complete the entire photocatalytic cycle.

## Conclusions

In summary, we have developed a novel method and strategy to achieve multilevel regulation of MOFs by integrating PSP and OMSs to comprehensively improve the overall performance for photocatalytic CO_2_ reduction. An unprecedented partitioned-*stp* MOF has been constructed based on atomically precise design and symmetry matching. This method preserves the OMSs while performing PSP, generating a new understanding of PSP and broadening its family of materials. The successful realization of the PSP strategy not only improves the pore structure but also enhances the network rigidity, which enabled NiTCPE-*pstp* to demonstrate enhanced structural stability and improved adsorption performance as an ideal catalytic platform. In addition, the synergistic effect between the main framework and the partitioning ligands endows NiTCPE-*pstp* with the special electronic structure and photoelectric activity, leading to a wider absorption range, enhanced electronic conductivity and matching potential, and efficient separation and transfer of photogenerated carriers. The multiple regulation of pore partition, microenvironment, and electronic structure effectively reduces the reaction energy barrier to promote the photocatalytic activity with superior CO generation rate, ultrahigh CO/H_2_ ratio, and nearly 100% selectivity. This work represents the first example of PSP-enabled MOF photocatalyst with multilevel structure and performance optimization. Such new atomically precise PSP strategy not only provides a promising method for postsynthesis modification of MOF materials but also provides a new perspective and model for the design and synthesis of efficient MOF photocatalysts as well as other catalytic materials.

## Supporting Information Available

All the experimental details; crystallographic data; ^1^H NMR and FT-IR spectra; TGA curves; PXRD patterns; UV–vis absorption spectrum; Tauc plots; Mott–Schottky plots; SEM images; HR-TEM images; steady-state PL emission spectra; EIS Nyquist plots; CCDC of 2172341–2172343 for TPAPA, NiTCPE-*stp*, and NiTCPE-*pstp*, respectively (PDF)
