# Supplementary material for: Multilevel-Regulated Metal–Organic Framework Platform Integrating Pore Space Partition and Open-Metal Sites for Enhanced CO2 Photoreduction to CO with Nearly 100% Selectivity
Source: J Am Chem Soc. 2023 Dec 6;145(50):27728–39. doi: 10.1021/jacs.3c10090 (PMC10739999; doi:10.1021/jacs.3c10090)
Supplement: Supplementary file 1 — ja3c10090_si_001.pdf [file ja3c10090_si_001.pdf]

Supporting Information

Multilevel-Regulated Metal-Organic Framework Platform Integrating Pore Space Partition and Open-Metal Sites for Enhanced CO_2 Photoreduction to CO with Nearly 100% Selectivity

Hui-Li Zheng,[†,‡] Jian-Qiang Zhao,[†,‡] Ya-Yong Sun,[†] An-An Zhang,[†] Yu-Jia Cheng,[†] Liang He,[†] Xianhui Bu,[§]* Jian Zhang,[†]* and Qipu Lin[†]*

[†] *State Key Laboratory of Structural Chemistry, Fujian Institute of Research on the Structure of Matter, Chinese Academy of Sciences, Fuzhou, 350002, China*

[‡] *University of Chinese Academy of Sciences, Beijing 100049, China*

[§] *Department of Chemistry and Biochemistry, California State University, Long Beach, California 90840, United States*

Table of Contents	
**Experimental**	Synthetic procedure and equipment.
**Figure S1**	The portion of 1D nanosized channels of NiTCPE-*stp* along *c*-axis.
**Figure S2**	The view of rhombic-like windows of NiTCPE-*stp* along *a*-axis.
**Figure S3**	The size- and symmetry-matching between 1D nanosized channels of NiTCPE-*stp* and TPAPA ligand.
**Figure S4**	The portion of 1D nanosized channels of NiTCPE-*pstp* with half of the metal sites for PSP.
**Figure S5**	The FT-IR spectra of compounds NiTCPE-*stp*, NiTCPE-*pstp* and TPAPA.
**Figure S6**	The structure of cage-like cavities with the size of *ca.* 21.2 × 21.2 × 9.1 $Å^3$ in NiTCPE-*pstp* after PSP.
**Figure S7**	The high-resolution XPS spectra of Ni 2p of NiTCPE-*stp* and NiTCPE-*pstp*.
**Figure S8**	The ^1H NMR spectra of activated NiTCPE-*stp* and NiTCPE-*pstp* digested in a mixture of DMSO-d_6 and DCl in D_2O.
**Figure S9**	The simulated and as-synthesized PXRD patterns of NiTCPE-*stp* and NiTCPE-*pstp*.
**Figure S10**	The TGA curves of NiTCPE-*stp* and NiTCPE-*pstp* under N_2 gas with a temperature range of room temperature to 800 °C.
**Figure S11**	The PXRD patterns of NiTCPE-*stp* after soaking in different solvents.
**Figure S12**	The PXRD patterns of NiTCPE-*pstp* after soaking in different organic solvents for a week.
**Figure S13**	The heats of adsorption (Q_{st}) of CO_2 for NiTCPE-*stp* and NiTCPE-*pstp*.
**Figure S14**	The UV–vis absorption spectrum of TPAPA.
**Figure S15**	The Tauc plots of NiTCPE-*stp* and NiTCPE-*pstp*.
**Figure S16**	The Mott–Schottky plots of NiTCPE-*stp*.
**Figure S17**	The photocatalytic CO evolution amounts for TPAPA.
**Figure S18**	The ^1H NMR spectrum of the liquid phase after the photocatalysis for NiTCPE-*pstp*.
**Figure S19**	The photocatalytic CO evolution amounts for NiTCPE-*pstp* and related control experiments.
**Figure S20**	The production amounts of CO and H_2 with NiTCPE-*stp* photocatalyst in recycling experiments.
**Figure S21**	The PXRD patterns of NiTCPE-*stp* before and after photocatalysis.

**Figure S22**	The N_2 uptake of NiTCPE-*stp* after photocatalysis at 77 K.
**Figure S23**	The production amounts of CO and H_2 with NiTCPE-*pstp* photocatalyst in recycling experiments.
**Figure S24**	The PXRD patterns of NiTCPE-*pstp* before and after photocatalysis.
**Figure S25**	The FT-IR spectra of NiTCPE-*pstp* before and after photocatalysis.
**Figure S26**	The XPS spectrum of NiTCPE-*pstp* after photocatalysis.
**Figure S27**	The SEM images of NiTCPE-*pstp* before and after photocatalysis.
**Figure S28**	The HR-TEM images of NiTCPE-*pstp* before and after photocatalysis.
**Figure S29**	The steady-state PL emission spectra of $[Ru(bpy)_3]Cl_2$ with the addition of NiTCPE-*stp*.
**Figure S30**	The steady-state spectra of $[Ru(bpy)_3]Cl_2$ upon the addition of increasing amounts of TIPA in the CH_3CN/H_2O solution.
**Figure S31**	The EIS Nyquist plots for NiTCPE-*stp* and NiTCPE-*pstp*.
**Figure S32**	*In situ* DRIFTS for CO_2 adsorption on NiTCPE-*pstp* in darkness at 5 min and 30 min.
**Figure S33**	The CO_2 adsorption energy for NiTCPE-*stp* and NiTCPE-*pstp*.
**Figure S34**	The configuration and adsorption energy of *COOH with C- or O-attaching to the Ni site for NiTCPE-*stp*.
**Figure S35**	The configuration and adsorption energy of *COOH with C- or O-attaching to the Ni site for NiTCPE-*pstp*.
**Figure S36**	The intermediate structures of photocatalytic CO_2 reduction to CO and H_2 production for NiTCPE-*stp*.
**Figure S37**	The intermediate structures of photocatalytic CO_2 reduction to CO and H_2 production for NiTCPE-*pstp*.
**Table S1**	Crystallographic data of TPAPA, NiTCPE-*stp* and NiTCPE-*pstp*.
**Table S2**	Selected bond lengths (Å) and angles (°) of NiTCPE-*stp*.
**Table S3**	Selected bond lengths (Å) and angles (°) of NiTCPE-*pstp*.
**Table S4**	The comparison of the photocatalytic performances of reported MOF-based photocatalysts for converting CO_2 to CO under visible light irradiation.

Experimental Procedures

Materials

All chemical reagents were used as purchased without further purification. $Ni(NO_3)_2·6H_2O$ and $[Ru(2,2'\text{-bipyridine})_3]Cl_2·6H_2O$ ($[Ru(bpy)_3]Cl_2$) were bought from Aladdin. *N,N*-dimethylacetamide (DMA), *N,N*-dimethylformamide (DMF), ethanol (EtOH), methanol (MeOH), pyridine (Py), formic acid, acetone, acetonitrile (MeCN), dichloromethane (CH_2Cl_2) and triisopropanolamine (TIPA) were bought from Sinopharm. Tetrakis(4-carboxyphenyl)ethylene (TCPE) were purchased from Alpha. Tris(4-aminophenyl)amine and 4-Pyridinecarboxaldehyde were purchased from Adamas. Carbon dioxide (CO_2, 99.999%) gas was supplied by Fuzhou Xinhang Industrial Gases Co., Ltd. The $^{13}CO_2$ was purchased from Wuhan New Reed Special Gas Co., Ltd. and its abundance is 99.4%.

Characterization

The thermogravimetric analysis (TGA) was performed using a Netzsch STA449C thermal analyzer under a constant flow of dry N_2 gas at a heating rate of 10 °C min^{-1} with a temperature range of room temperature to 800 °C. Elemental analyses (C, H, N) were performed on a Vario EL-Cube. Fourier transform infrared (FT-IR) spectra were recorded from KBr pellets containing 1% of the compound in the range of 400~4000 cm^{-1} on a Nicolet Magna 750 FT-IR spectrometer. Powder X-ray diffraction (PXRD) analyses were recorded on a Rigaku Dmax2500 diffractometer with Cu *Kα* radiation (λ = 1.54056 Å). Surface chemical analyses were performed by X-ray photoelectron spectroscopy (XPS, Thermo Fisher, ESCALAB 250Xi). Ultraviolet-visible (UV-Vis) diffuse-reflectance spectra (DRS) were performed on a Shimadzu UV-1201PC spectrophotometer.

Scanning electron microscopy (SEM) images were obtained by a Zeiss Sigma 500. Transmission Electron Microscope (TEM) images were recorded by FEI Tecnai G2 f20 s-twin 200kV. The CO_2 adsorption-desorption isotherms were performed on a Micromeritics ASAP 2020 surface area at 273 K. The generated gas products were analyzed by a gas chromatography analyzer (FULI 9790II) equipped with the flame ionization detector (FID) and thermal conductivity detector (TCD). The filtrate of the reaction was measured by NMR spectroscopy (ECZ400S, AVANCE III). The mass spectral of ^{13}CO were measured using a chromatography-mass spectrometry (Agilent, 7890B). *In-situ* electron paramagnetic resonance (EPR) spectra was measured by a Bruker A300 instrument.

Synthesis of TPAPA:

Tris(4-aminophenyl)amine (2.9 g, 10 mmol) was added to a mixed solution of 100 mL of methanol and ethanol. Under stirring conditions, excess 4-Pyridinecarboxaldehyde (3.3 mL, 35 mmol) was added drop by drop to the above mixture solution. After stirring overnight, the solution became a clear yellow-brown color. After heating and steaming, the remaining 20 mL of solution was placed in a refrigerator for one night. Yellow rhombic crystals of TPAPA were obtained. The yield was 87% based on Tris(4-aminophenyl)amine. ^1H NMR (400 MHz, DMSO-d_6) δ 8.76-8.74 (m, 9H), 7.85 (d, J = 6.0 Hz, 6H), 7.41 (d, J = 8.8 Hz, 6H), 7.15 (d, J = 8.8 Hz, 6H).

Synthesis of NiTCPE-*stp*:

$Ni(NO_3)_2{\cdot}6H_2O$ (60 mg, 0.2 mmol) and TCPE (35 mg, 0.07 mmol) were dissolved in a DMA-MeOH-H_2O solution (10:1:1, 6 mL) in a 20 mL vial. And then 5 drops pyridine and formic acid were added into above mixture solution, respectively. The vial was heated at 120 °C for 5 days and then cooled to room temperature. Green needle crystals of NiTCPE-*stp* were obtained. The yield of the NiTCPE-*stp* was 49 % based on TCPE.

Synthesis of NiTCPE-*pstp*:

$Ni(NO_3)_2 \cdot 6H_2O$ (60 mg, 0.2 mmol), TCPE (35 mg, 0.07 mmol) and TPAPA (45 mg, 0.08 mmol) were dissolved in a DMA-MeOH solution (10:1, 6 mL) in a 20 mL vial. And then 5 drops formic acid was added into above mixture solution. The vial was heated at 120 °C for 5 days and then cooled to room temperature. Dark red brown crystals of NiTCPE-*pstp* were obtained. The yield of the NiTCPE-*stp* was 53 % based on TCPE.

X-ray Crystallography

The single-crystal X-ray diffraction (XRD) analyses of compounds TPAPA and NiTCPE-*pstp* were collected on ROD, Synergy Custom system, HyPix diffractometer with micro-focus metaljet *Ka* (λ = 1.34050 Å) radiation at 111 and 100 K, respectively. The XRD data of NiTCPE-*stp* were collected on a Bruker Smart Apex CCD diffractometer with graphite monochromatic Mo-*Ka* radiation (λ = 0.71073 Å) at 293 K. The crystal structures were solved and refined by full matrixcs methods against F^2 using SHELXL-2014 program package and Olex-2 software.[1-2] All non-hydrogen atoms were refined with anisotropic temperature parameters and hydrogen positions were fixed at calculated positions and refined isotropically. Crystal data and structure refinements for TPAPA, NiTCPE-*stp* and NiTCPE-*pstp* are listed in Table S1. The selected bond lengths and angles of NiTCPE-*stp* and NiTCPE-*pstp* are listed in Table S2. The crystal structures of three compounds have been deposited at the CCDC, and the CCDC numbers are 2172341, 2172342 and 2172343 for TPAPA, NiTCPE-*stp* and NiTCPE-*pstp*, respectively.

Photocatalytic Reaction

The photocatalytic CO_2 reduction experiments were performed in a sealed Pyrex reactor (250 mL) with a top flat quartz window for light irradiation and a silicone rubber septum was fixed on its side

for sampling produced gaseous products in the headspace of reaction cell. Catalyst (5 mg) and $[Ru(bpy)_3]Cl_2$ (0.05 mmol) were added into the mixed solution of CH_3CN/H_2O (4:1) containing 10% TIPA with magnetic stirring and the total amount of solution was 60 mL. After degassing with CO_2 to remove dissolved air for 20 minutes, the reaction was performed under the irradiation of a 300 W Xe lamp with UV and IR-cut to keep the wavelengths in the range from 420 to 800 nm. The reaction temperature was maintained at 298 K by using cooling water circulation. In order to detect the content of carbon monoxide produced by the reaction mixture, 100 μL of gas-product was extracted from the reactor with a syringe and injected into the gas chromatograph with a FID detector, using argon as the carrier gas and reference gas. To detect the formation of H_2 from the reaction mixture, 100 μL of gas-product was taken out with a syringe and injected into a GC with a TCD detector, using argon as the carrier gas and reference gas. By comparing the integrated area of the gas-phase product with the calibration curve, the volume of CO_2 and H_2 can be calculated. All photocatalytic reactions were repeated three times to ensure the accuracy of the experimental data. The recycled experiments for photocatalytic CO evolution reaction were performed under the same conditions to evaluate the stability and reusability of the catalyst in a long-term operation and the photocatalyst after each test was recovered by centrifugal washing.

Photoluminescent quenching Measurements

Steady-state photoluminescence (PL) spectra and time-resolved PL decay spectra were performed on a FLS1000 Spectrometer. The photoluminescent quenching of $[Ru(bpy)_3]Cl_2$ (80 μM, 8 mL) were performed in the original reaction system (CH_3CN/H_2O = 4:1, with TIPA 10%) upon the addition of increasing amounts of catalyst (0, 0.2, 0.4, 0.6, 0.8 and 1.0 mg) and TIPA (0, 1.0, 2.0 mL), respectively. These samples were excited at λ_{ex} = 420 nm. The solution of $[Ru(bpy)_3]Cl_2$ (80 μM, 8

mL) before and after addition of NiTCPE-*stp*/ NiTCPE-*pstp* (1.00 mg) was used for time-resolved PL decay testing emission at λ_{em} = 600 nm.

Photo/electrochemical Measurements

The Mott-Schottky plots were measured on an IM 6 electrochemical system via a conventional three-electrode system in a 0.2 M Na_2SO_4 aqueous solution. 2 mg photocatalyst were dispersed in a mixed solution of 0.5 mL ethanol and 10 μL Nafion dispersion solutions to generate a homogeneous slurry, respectively. Subsequently, 30 μL of slurry was transferred and coated on fluoride-tin oxide (FTO) glass plates and covering approximately 0.25 cm^2, Pt plate as the counter electrode and Ag/AgCl as reference electrode at frequencies of 500, 1000, and 1500 Hz, respectively. Photocurrent density measurements were performed on an electrochemical workstation CHI 760E (Chenhua Instrumen, Shanghai, China) in a standard three-electrode electrochemical cell. The working electrodes were prepared as in Mott-Schottky experiments with the counter electrode of Pt plate, the reference electrode of Ag/AgCl. A 0.2 M of Na_2SO_4 solution was used as the electrolyte.

***In-situ* DRIFTS Measurements**

In-situ diffuse reflectance infrared Fourier transform spectroscopy (DRIFTS) was carried out using a Thermo Scientific Nicolet 6700 spectrometer. Each spectrum was obtained by averaging 128 scans, with a resolution of 4 cm^{-1}. CO_2 flow (20 mL/min) was bubbled into deionized water and then passed through the cell loading with a powder mixture of NiTCPE-*pstp* and $[Ru(bpy)_3]Cl_2$, with a lower concentration of TIPA. The *in-situ* DRIFTS measurements were conducted using a Praying Mantis DRIFTS accessory and a reactor, and a 300 W Xe lamp was connected to a liquid light guide for irradiation. In the process of recording spectra, the samples were purged with wet CO_2 for 30 min until their spectra in dark stabilized. Visible light irradiation was applied to the powder through a

quartz window in the sample cell, and spectra were recorded as a function of time to analyze the dynamic behavior of surface carbon contamination.

Computational Details

All DFT calculations were performed using the Vienna ab initio simulation package (VASP) in accordance with first principles.[3-5] Spin-polarization density functional theory (DFT) calculations were carried out using the Perdew-Burke-Ernzerhof (PBE) formulation within the generalized gradient approximation (GGA).[3-5] The projected augmented wave (PAW) potentials were used to accurately describe the ionic cores and consider valence electrons. A plane wave basis set with a kinetic energy cutoff of 520 eV was employed.[6-7] The GGA+U method was implemented, with an effective Hubbard U value set at 4.814 eV for Ni. The Gaussian smearing method with a width of 0.05 eV was used to evaluate partial occupancies of the Kohn–Sham orbitals. The electronic energy was considered self-consistent when the energy change was smaller than 10^{-5} eV. The geometry optimization was considered convergent when the energy change was smaller than 0.05 eV $Å^{-1}$. The U correction is used for Ni atoms in the structure. For the Brillouin zone integration, a 1×1×1 Monkhorst-Pack k-point sampling was performed. In the HOMO-LUMO calculation, a K-point grid of 2×2×2 was initially used to calculate the band structure, and the post-processing process was visualized with the help of VASPKIT software. Subsequently, the adsorption energies (E_{ads}) were calculated as the equation: $E_{ads} = E_{ad/sub} - E_{ad} - E_{sub}$, where $E_{ad/sub}$, E_{ad}, and E_{sub} represent the total energies of the optimized adsorbate/substrate system, the adsorbate in the structure, and the clean substrate, respectively. The calculation of the free energy involves the equation: $G = E_{ads} + ZPE - TS$, where G represents the free energy, Eads is the total energy obtained from DFT calculations, ZPE is the zero-point energy, and TS represents the entropic contributions (T = 300 K).

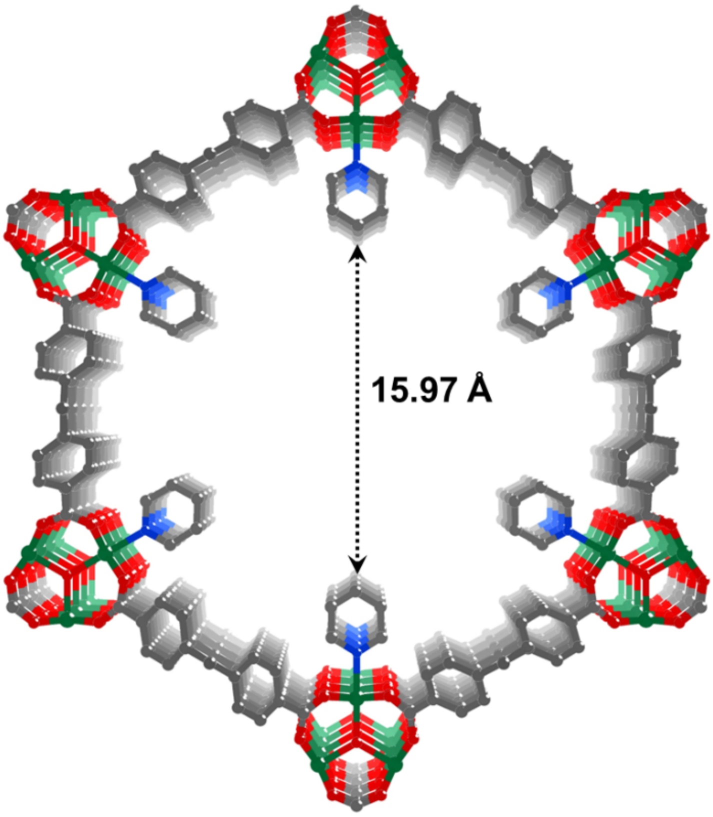

Figure S1 The portion of 1D nanosized channels of NiTCPE-*stp* along *c*-axis.

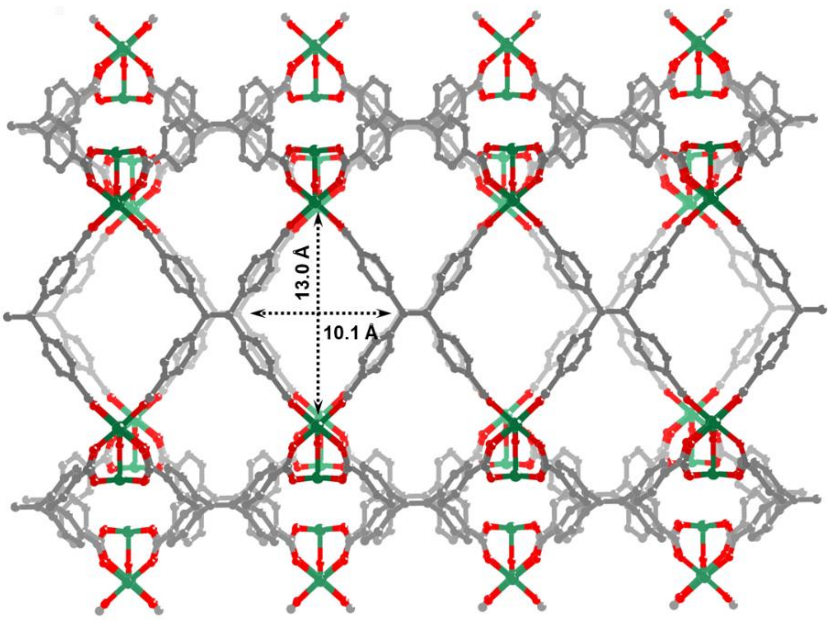

Figure S2 The view of rhombic-like windows of NiTCPE-*stp* along *a*-axis.

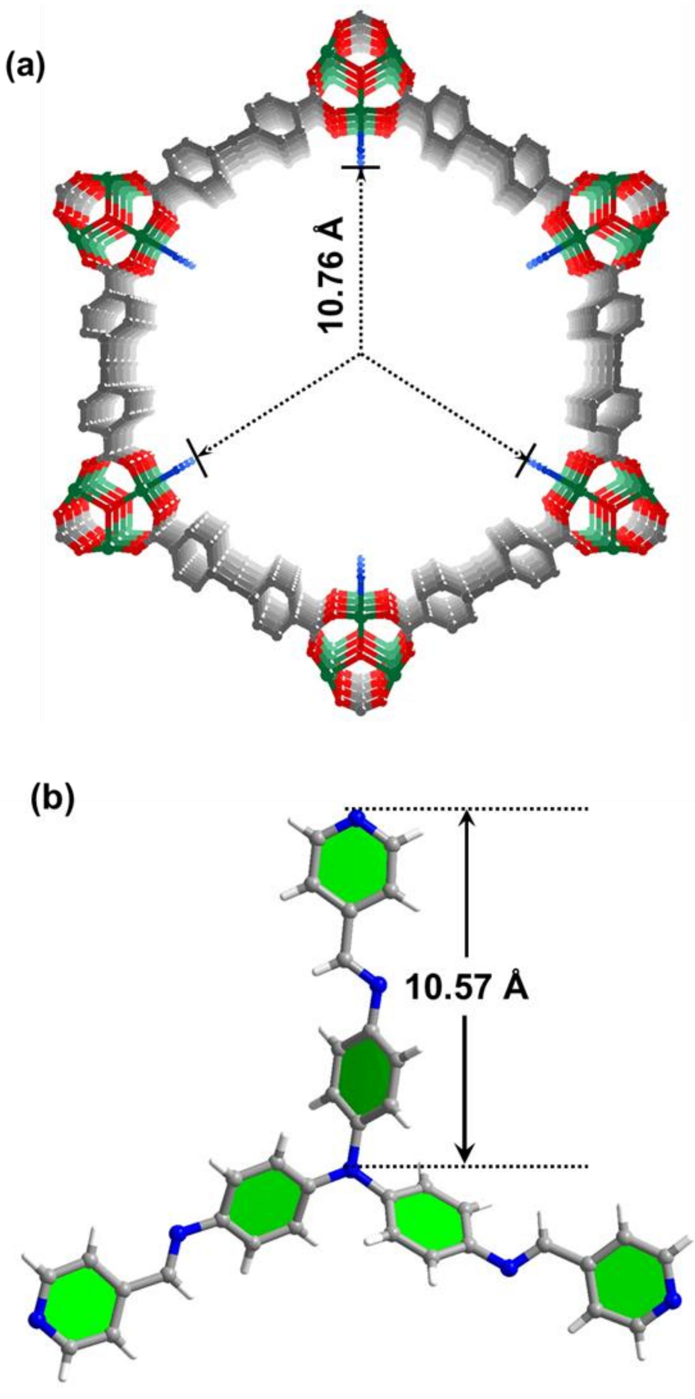

Figure S3 The size- and symmetry-matching between 1D nanosized channels of NiTCPE-*stp* and TPAPA ligand.

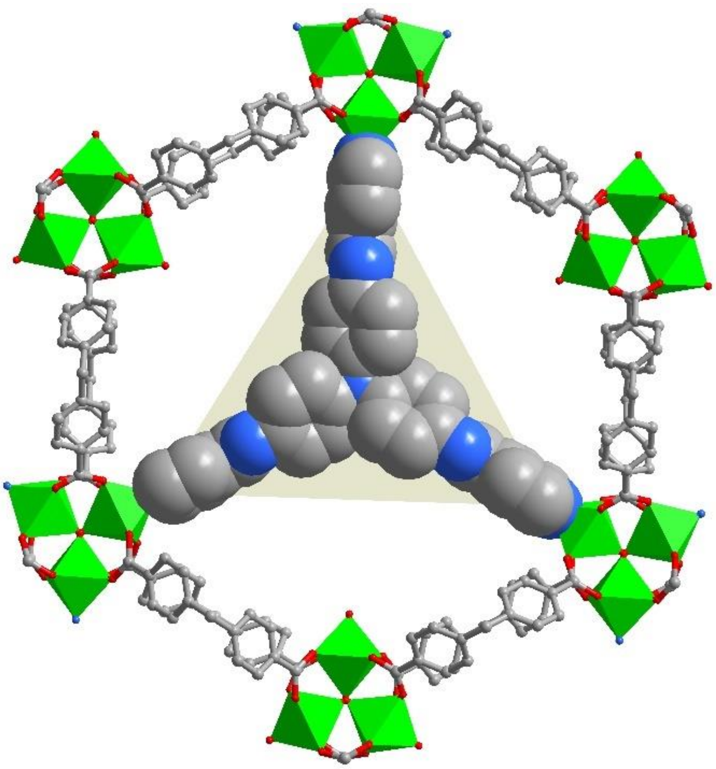

Figure S4 The portion of 1D nanosized channels of NiTCPE-*pstp* with half of the metal sites for PSP.

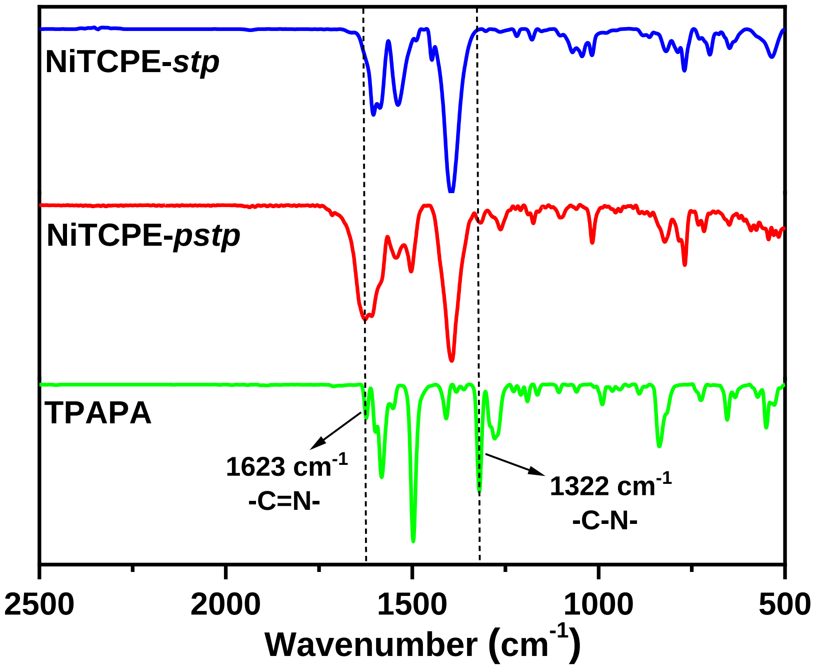

Figure S5 The FT-IR spectra of compounds NiTCPE-*stp*, NiTCPE-*pstp* and TPAPA.

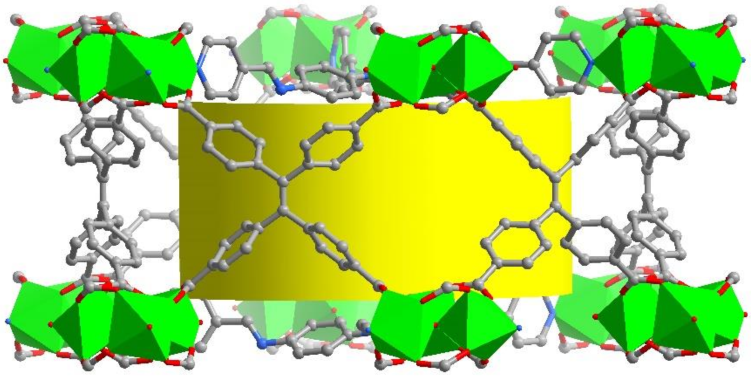

Figure S6 The structure of cage-like cavities with the size of *ca.* 21.2 × 21.2 × 9.1 Å^3 in NiTCPE-*pstp* after PSP.

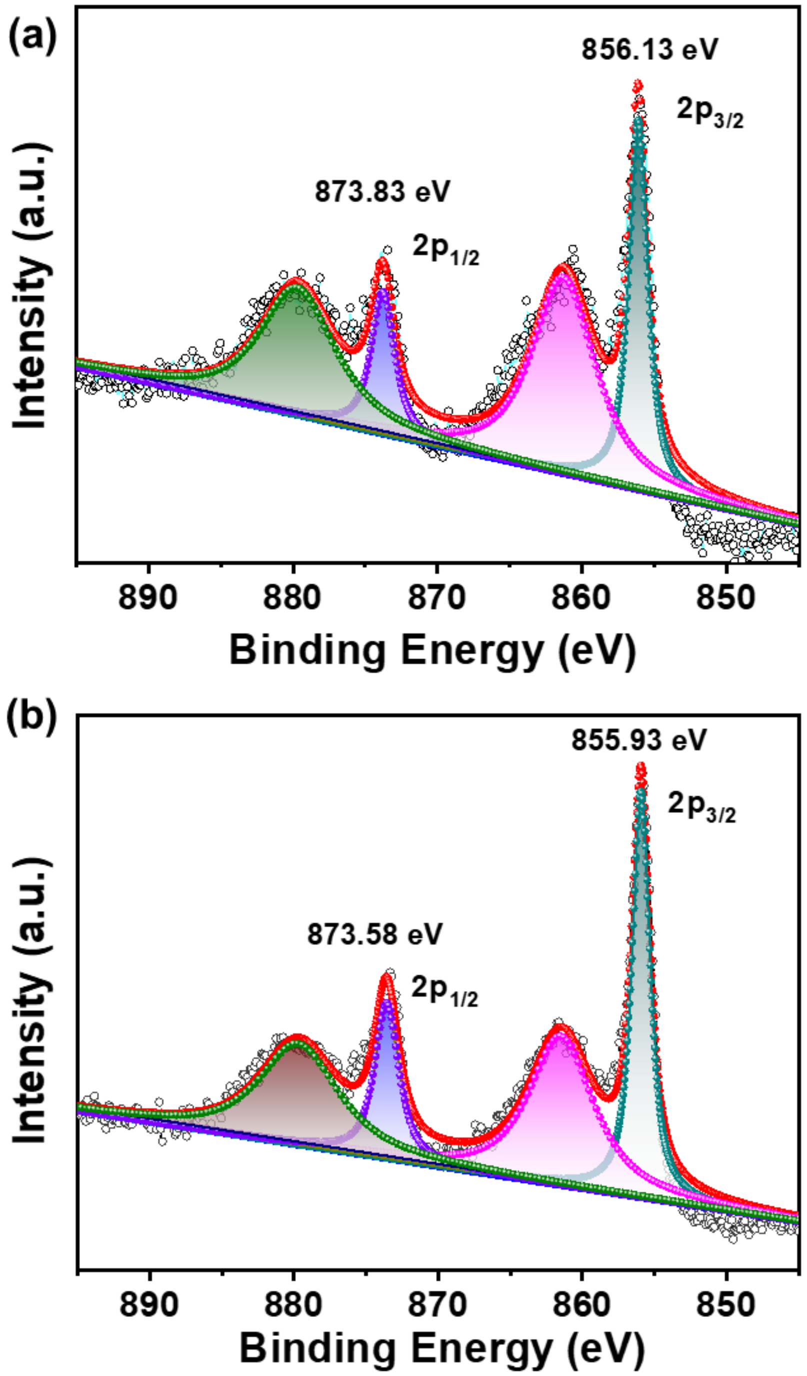

Figure S7 The high-resolution XPS spectra of Ni 2p of (a) NiTCPE-*stp* and (b) NiTCPE-*pstp*.

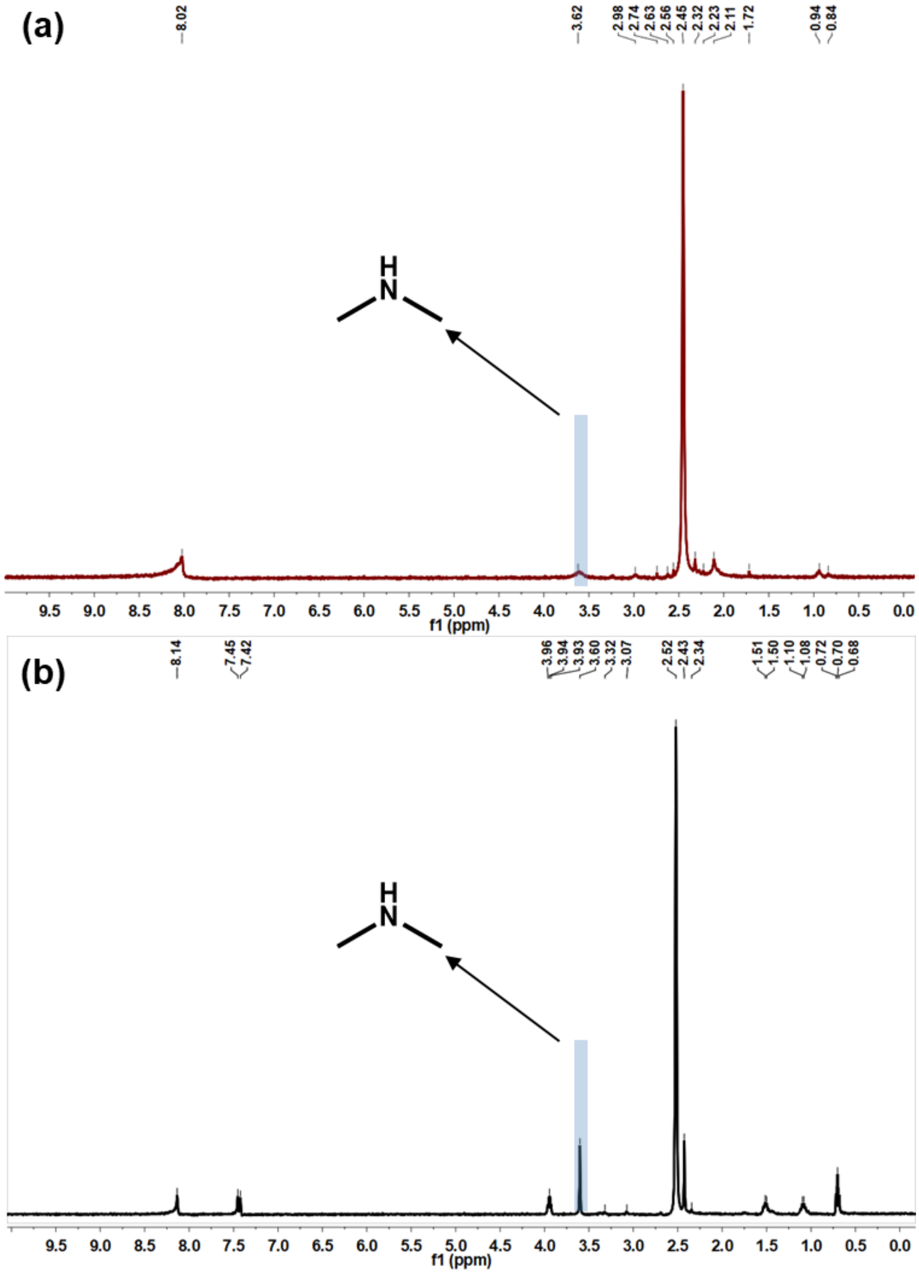

Figure S8 The ^{1}H NMR spectra of activated (a) NiTCPE-*stp* and (b) NiTCPE-*pstp* digested in a mixture of DMSO-d_6 and DCl in D_2O.

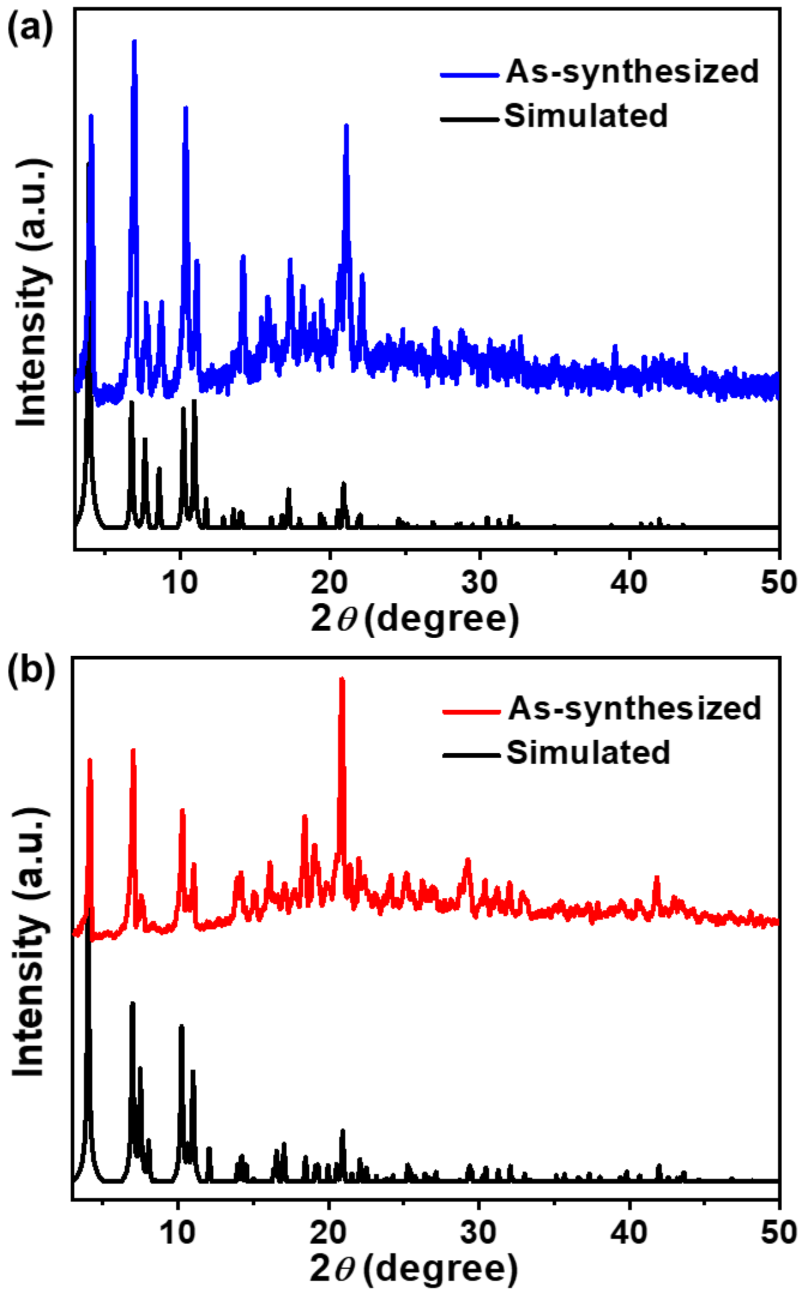

Figure S9 The simulated and as-synthesized PXRD patterns of (a) NiTCPE-*stp* and (b) NiTCPE-*pstp*.

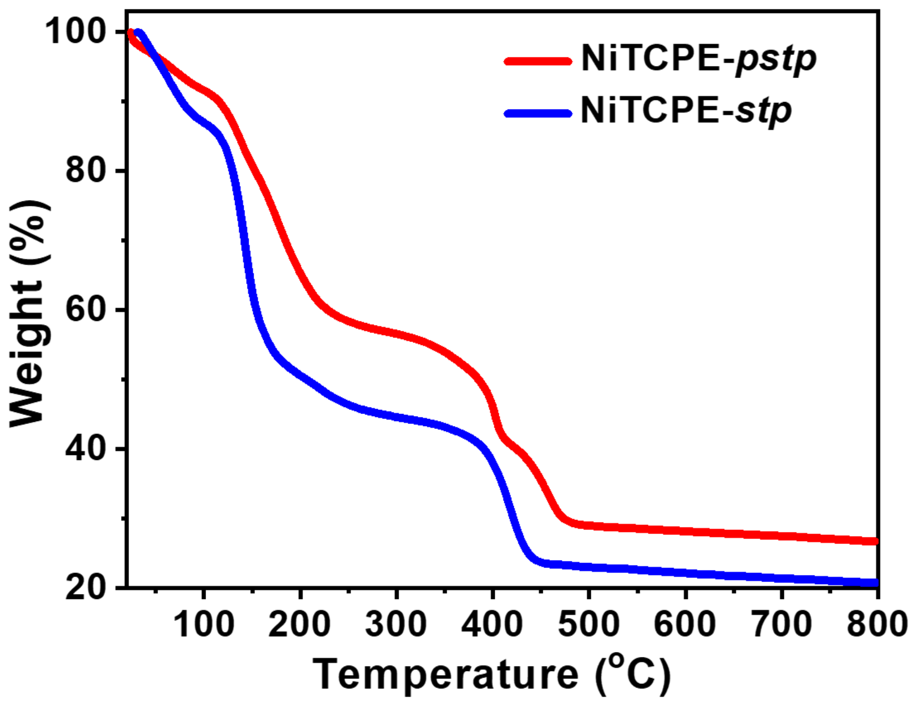

Figure S10 The TGA curves of NiTCPE-*stp* and NiTCPE-*pstp* under N_2 gas with a temperature range of room temperature to 800 °C.

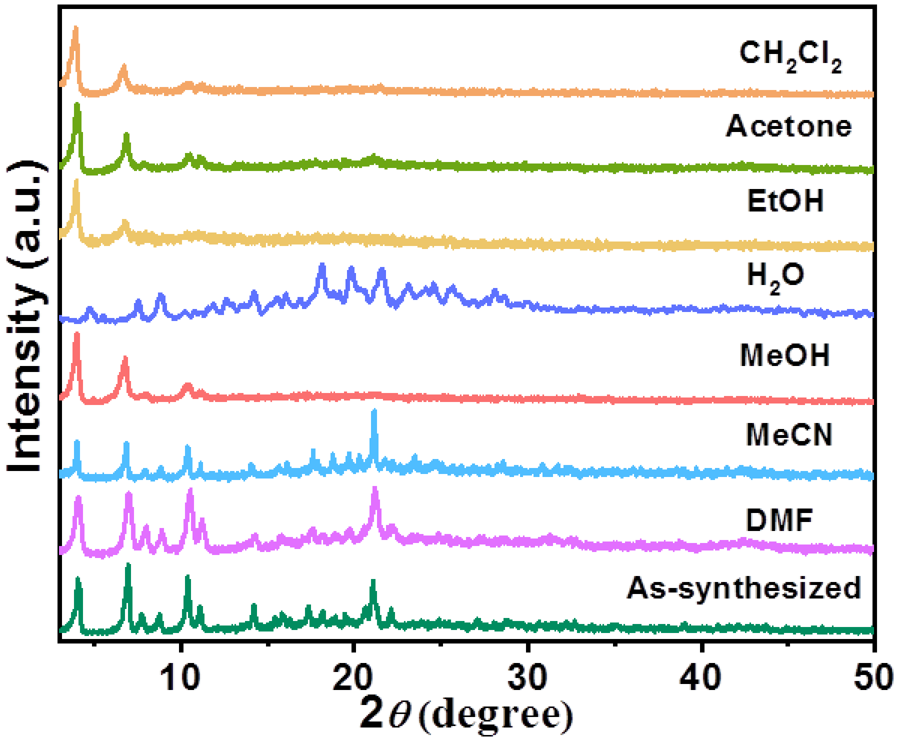

Figure S11 The PXRD patterns of NiTCPE-*stp* after soaking in different solvents.

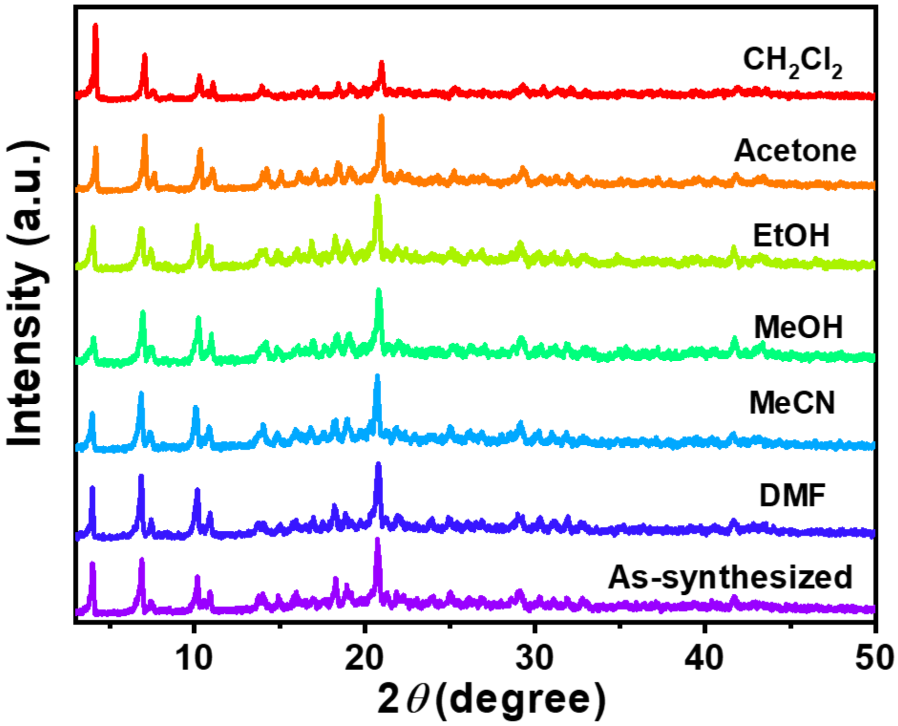

Figure S12 The PXRD patterns of NiTCPE-*pstp* after soaking in different organic solvents for a week.

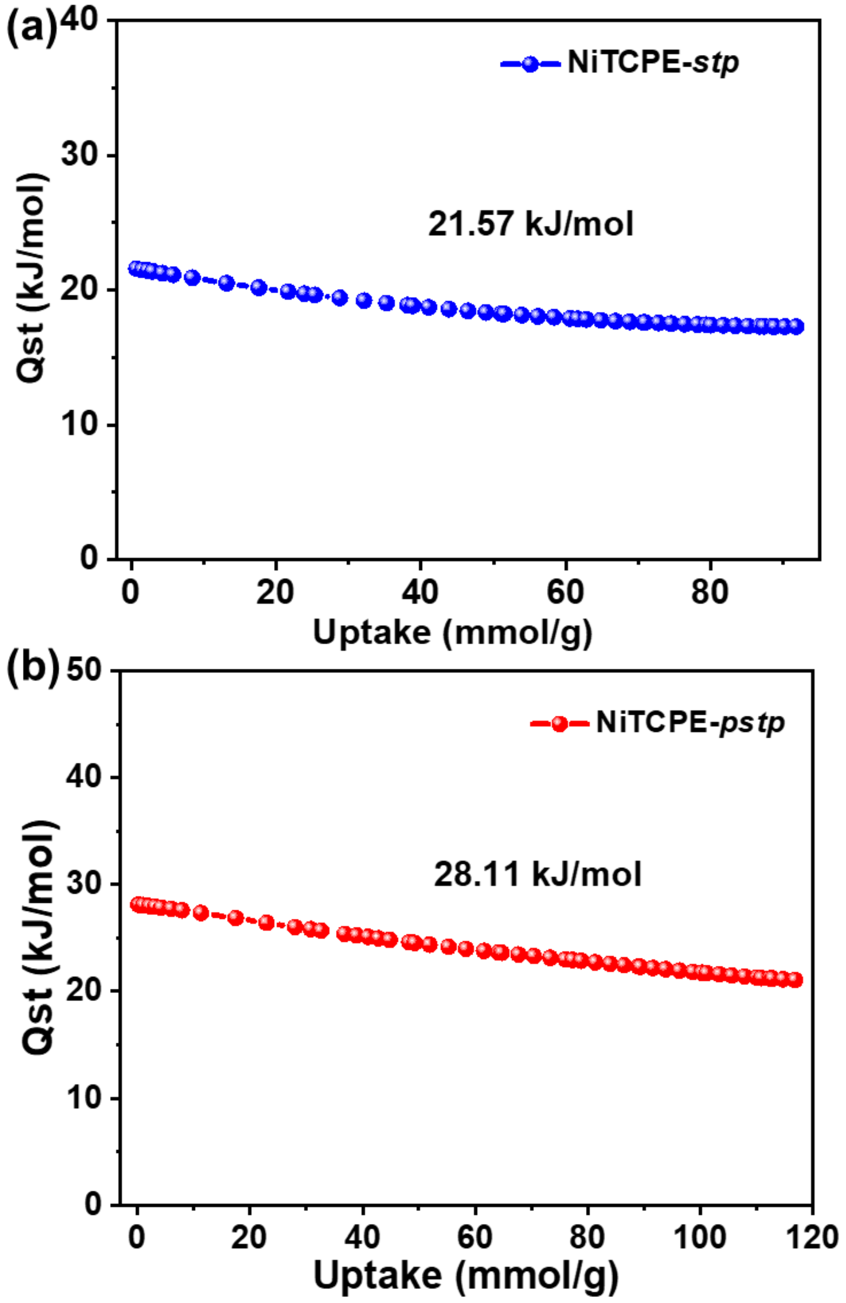

Figure S13 The heats of adsorption (Q_{st}) of CO_2 for (a) NiTCPE-*stp* and (b) NiTCPE-*pstp*.

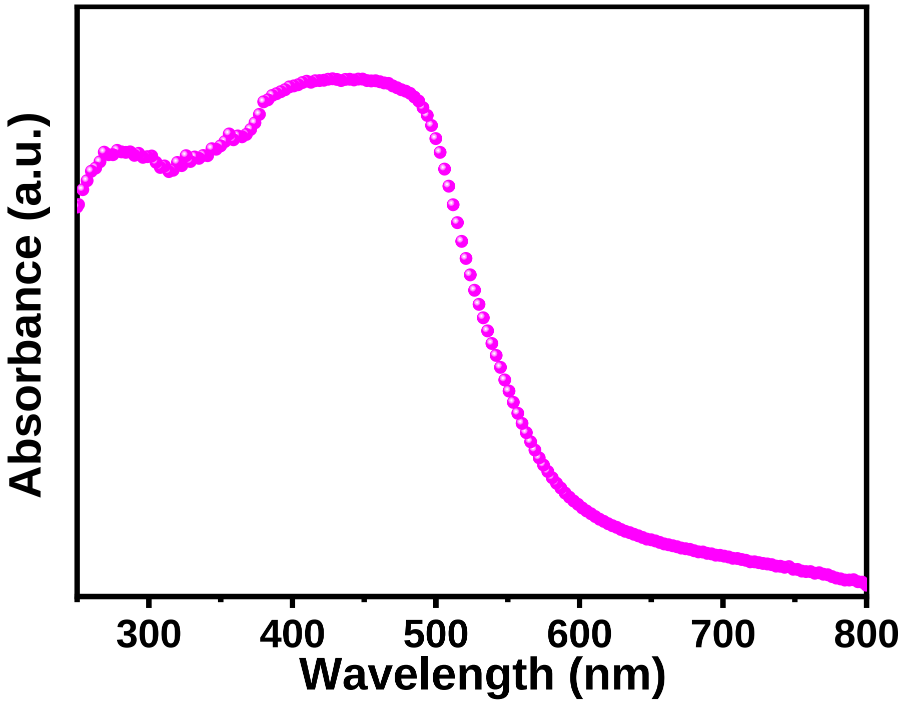

Figure S14 The UV–vis absorption spectrum of TPAPA.

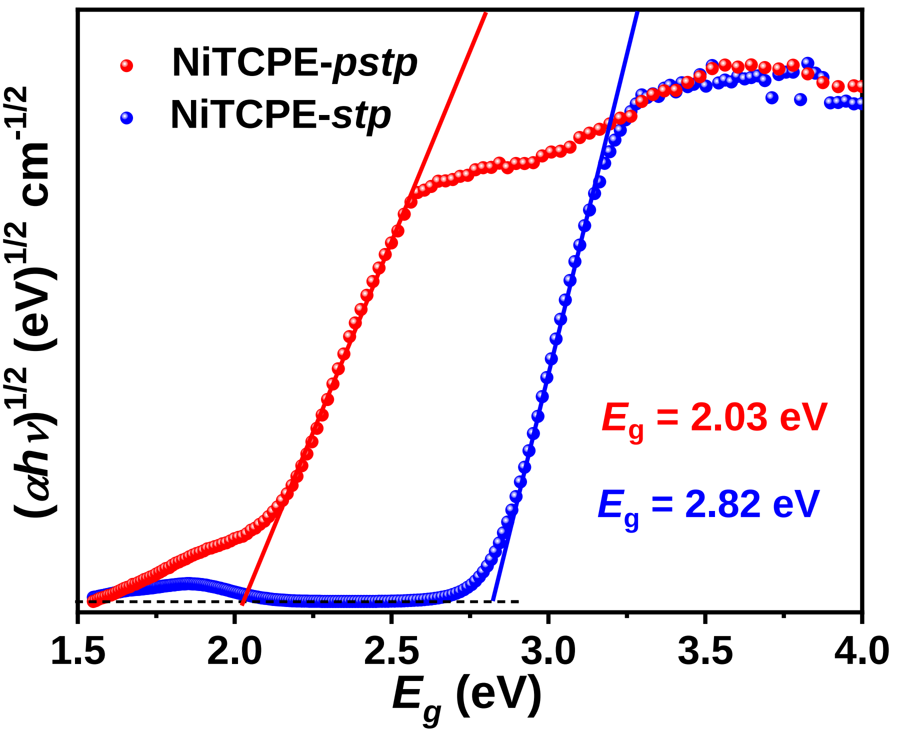

Figure S15 The Tauc plots of NiTCPE-*stp* and NiTCPE-*pstp*.

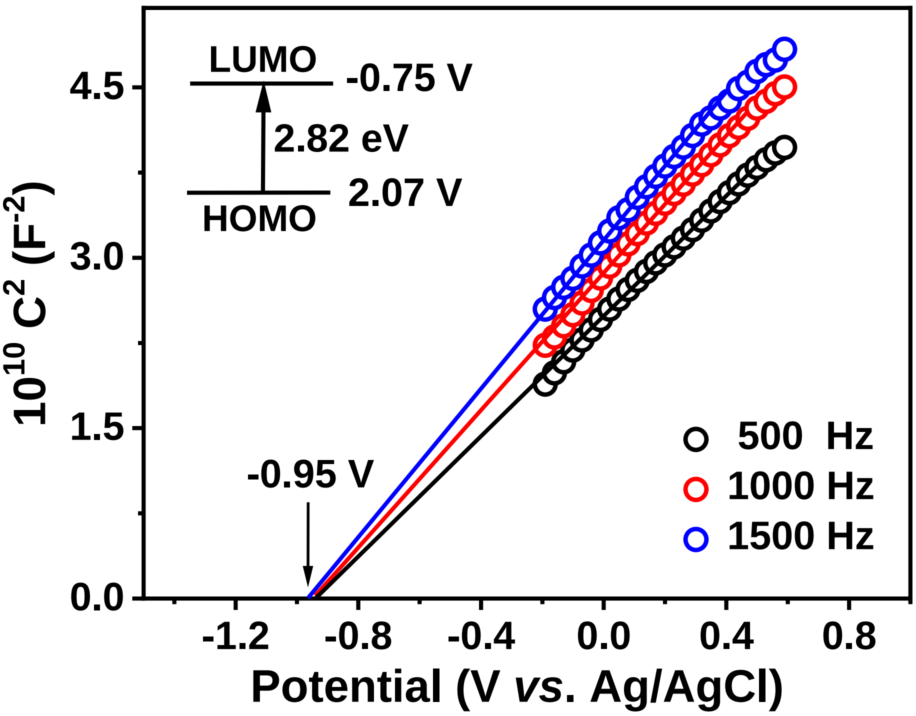

Figure S16 The Mott–Schottky plots of NiTCPE-*stp*.

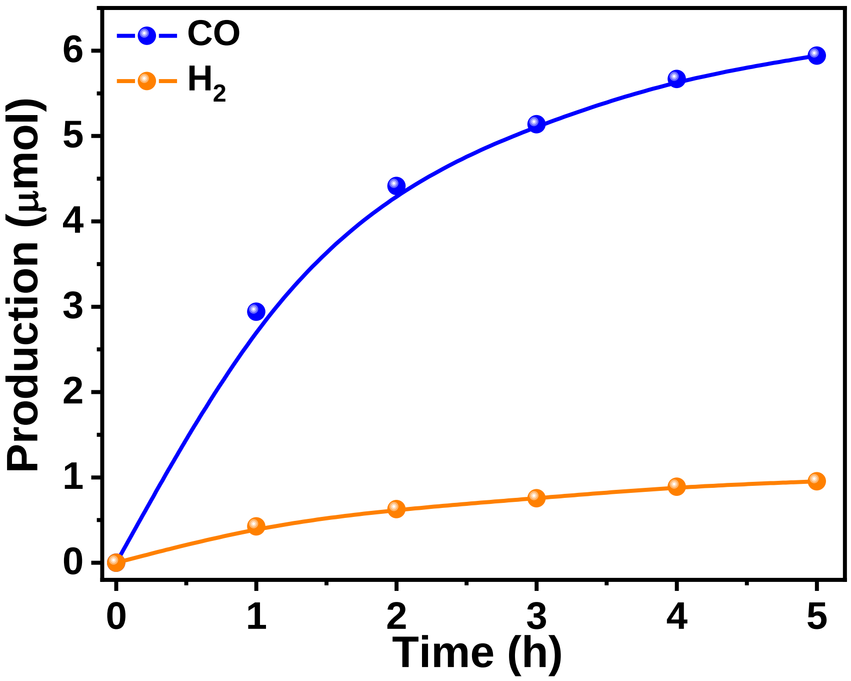

Figure S17 The photocatalytic CO evolution amounts for TPAPA.

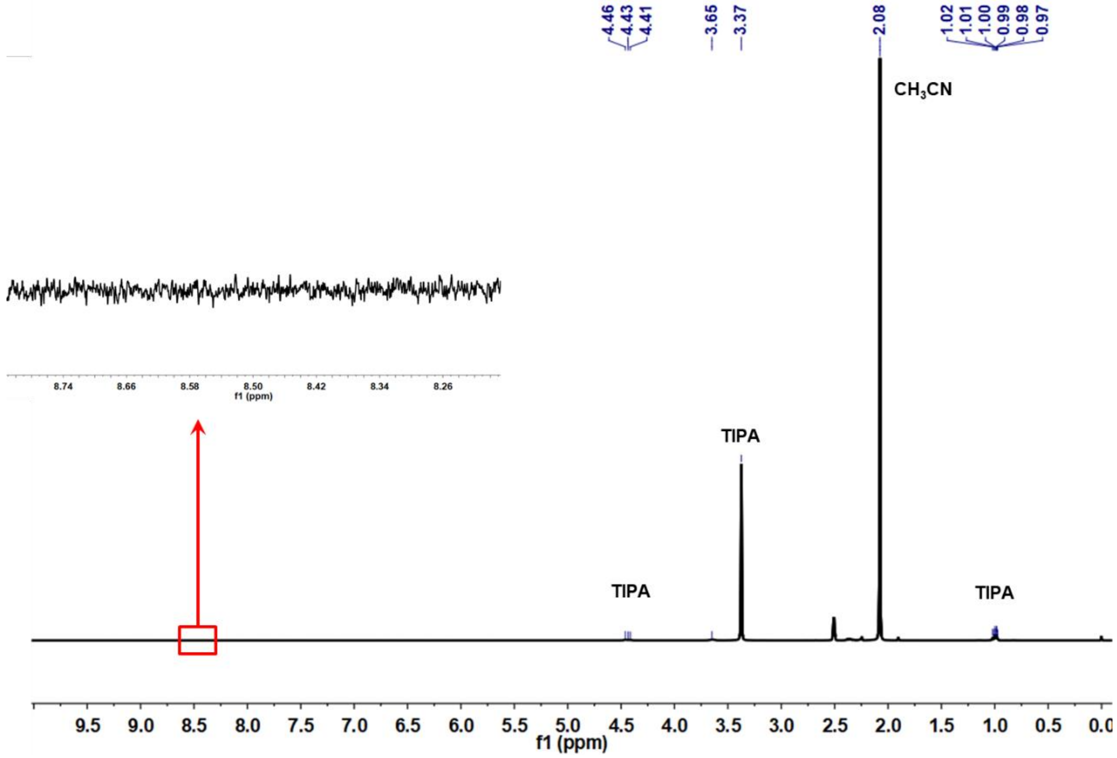

Figure S18 The ^{1}H NMR spectrum of the liquid phase after the photocatalysis for NiTCPE-*pstp*.

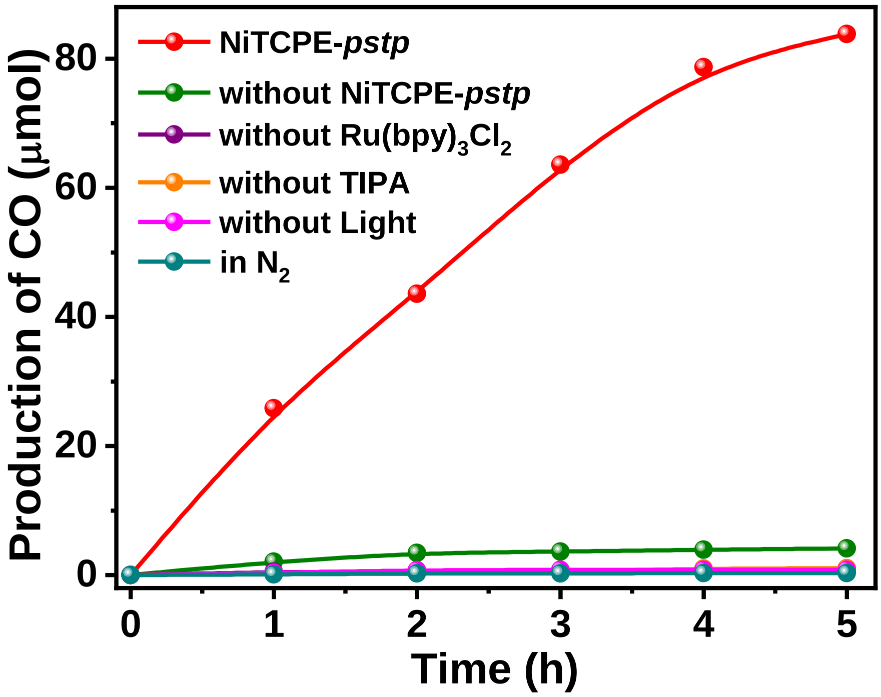

Figure S19 The photocatalytic CO evolution amounts for NiTCPE-*pstp* and related control experiments.

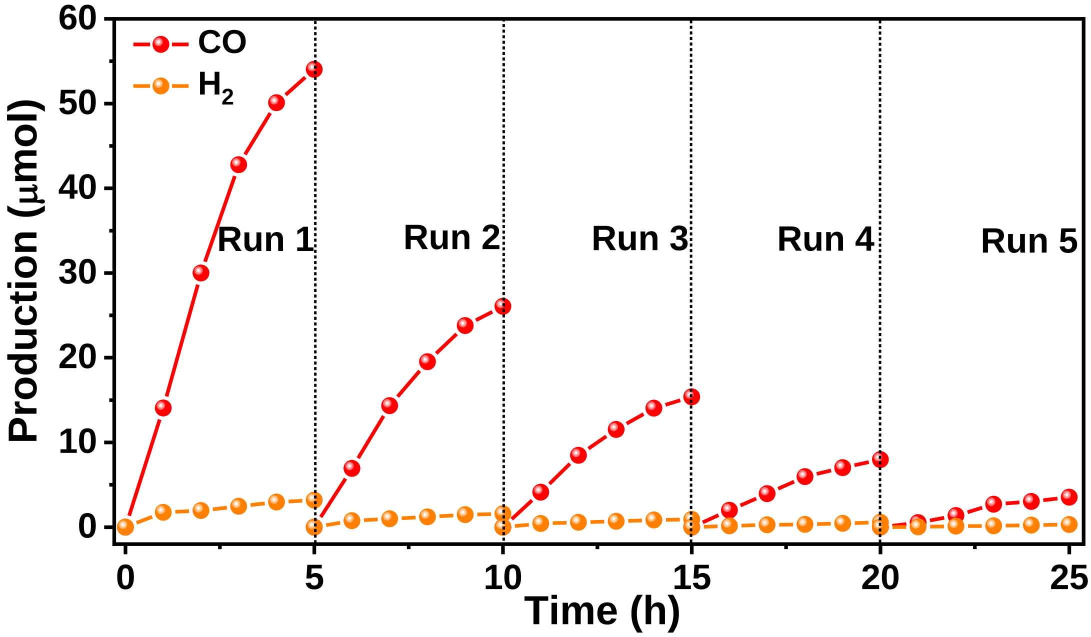

Figure S20 The production amounts of CO and H_2 with NiTCPE-*stp* photocatalyst in recycling experiments.

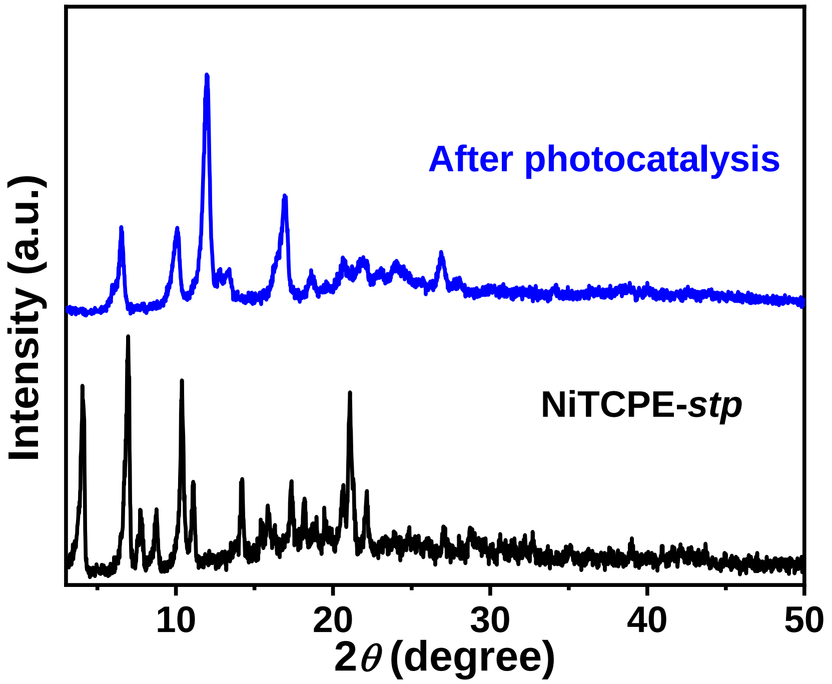

Figure S21 The PXRD patterns of NiTCPE-*stp* before and after photocatalysis.

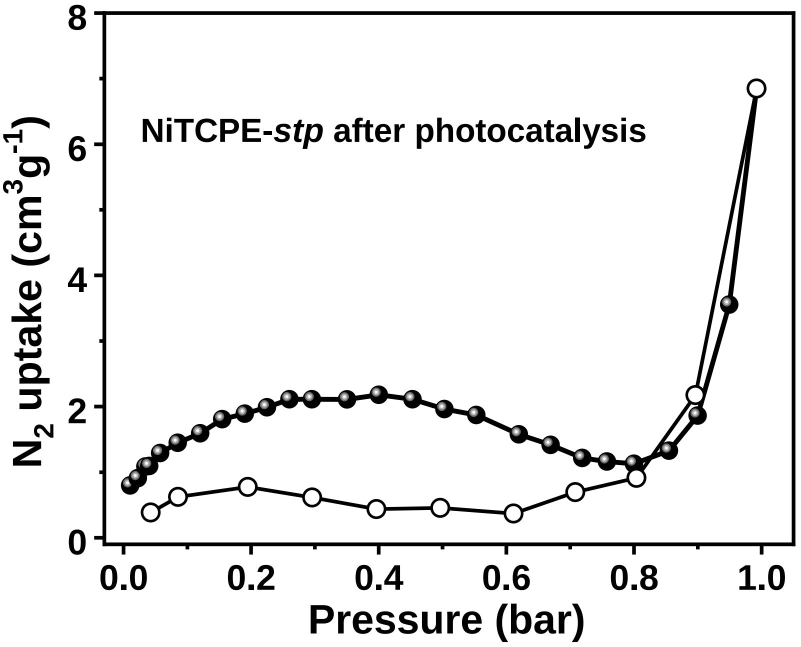

Figure S22 The N_2 uptake of NiTCPE-*stp* after photocatalysis at 77 K.

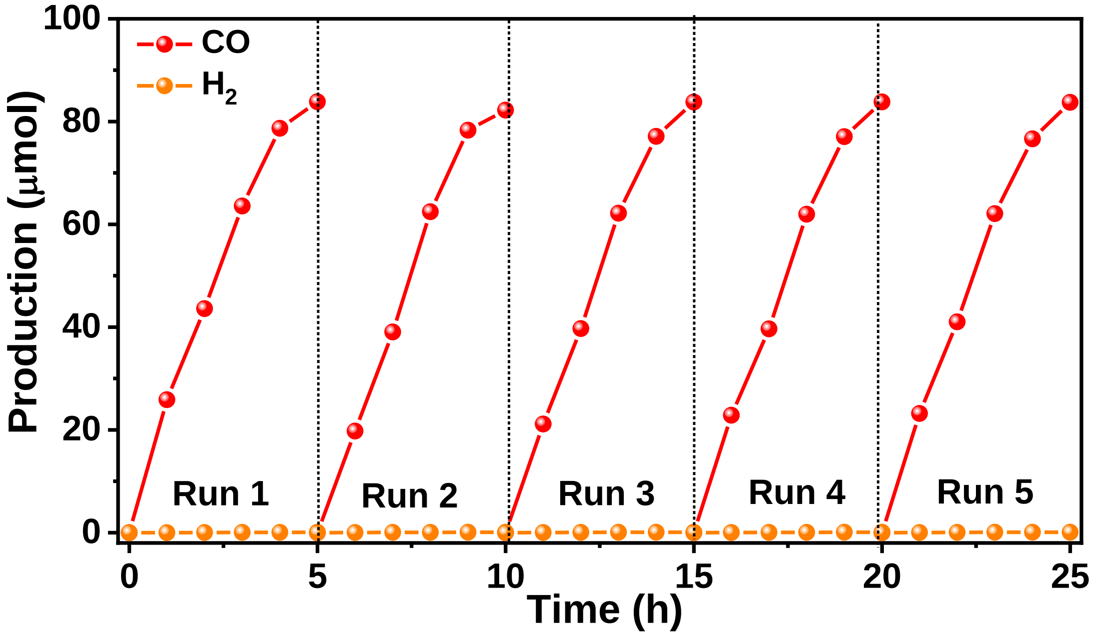

Figure S23 The production amounts of CO and H_2 with NiTCPE-*pstp* photocatalyst in recycling experiments.

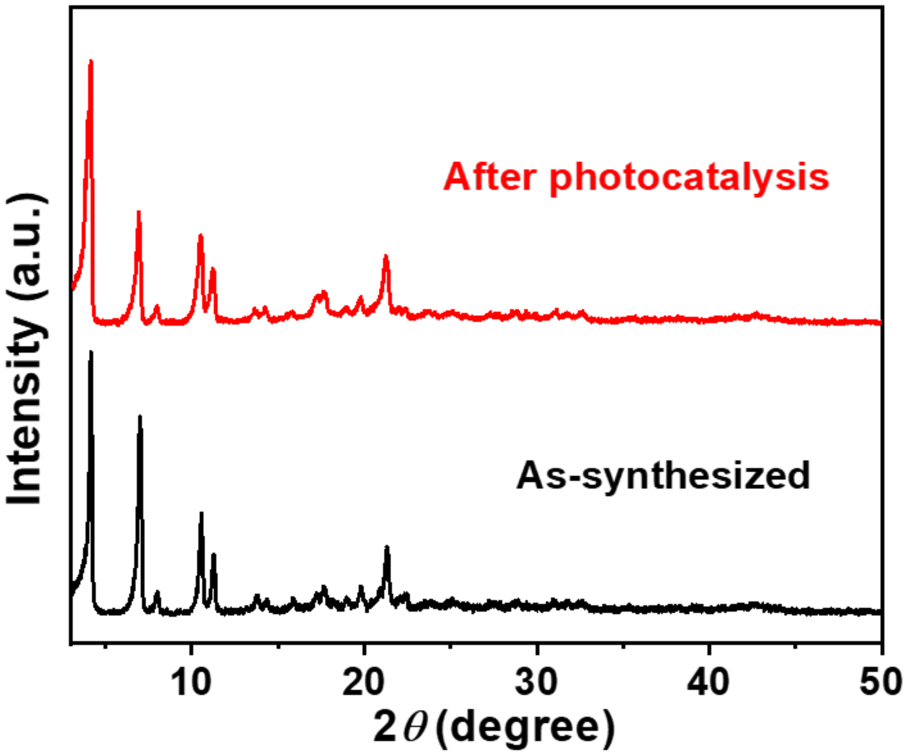

Figure S24 The PXRD patterns of NiTCPE-*pstp* before and after photocatalysis.

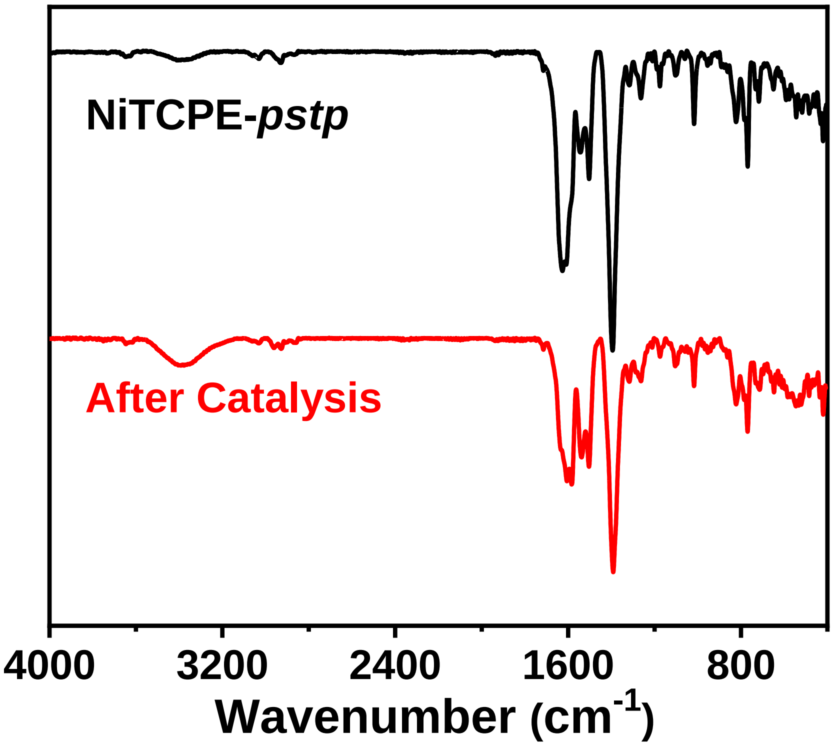

Figure S25 The FT-IR spectra of NiTCPE-*pstp* before and after photocatalysis.

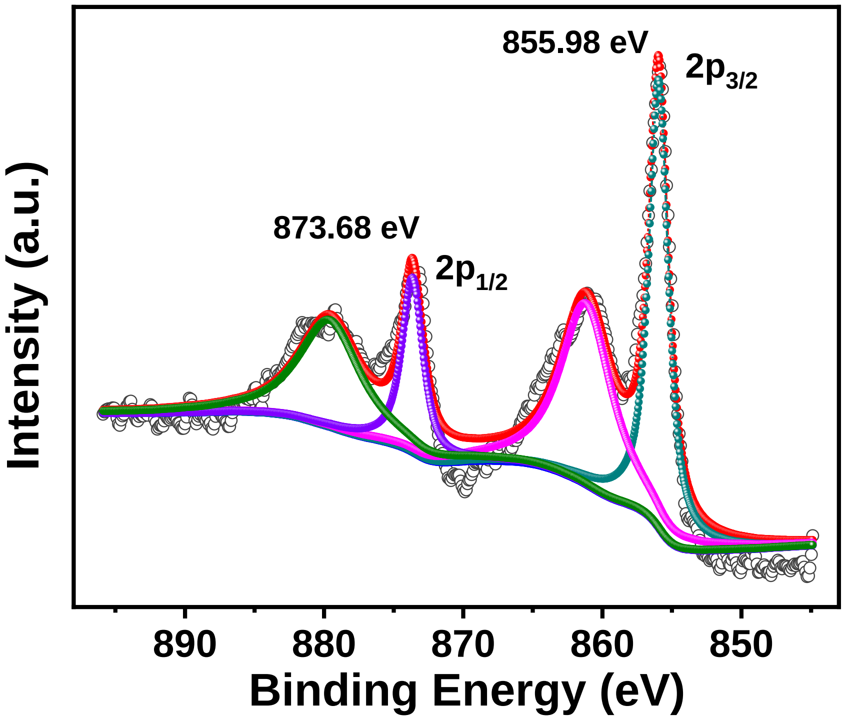

Figure S26 The XPS spectrum of NiTCPE-*pstp* after photocatalysis.

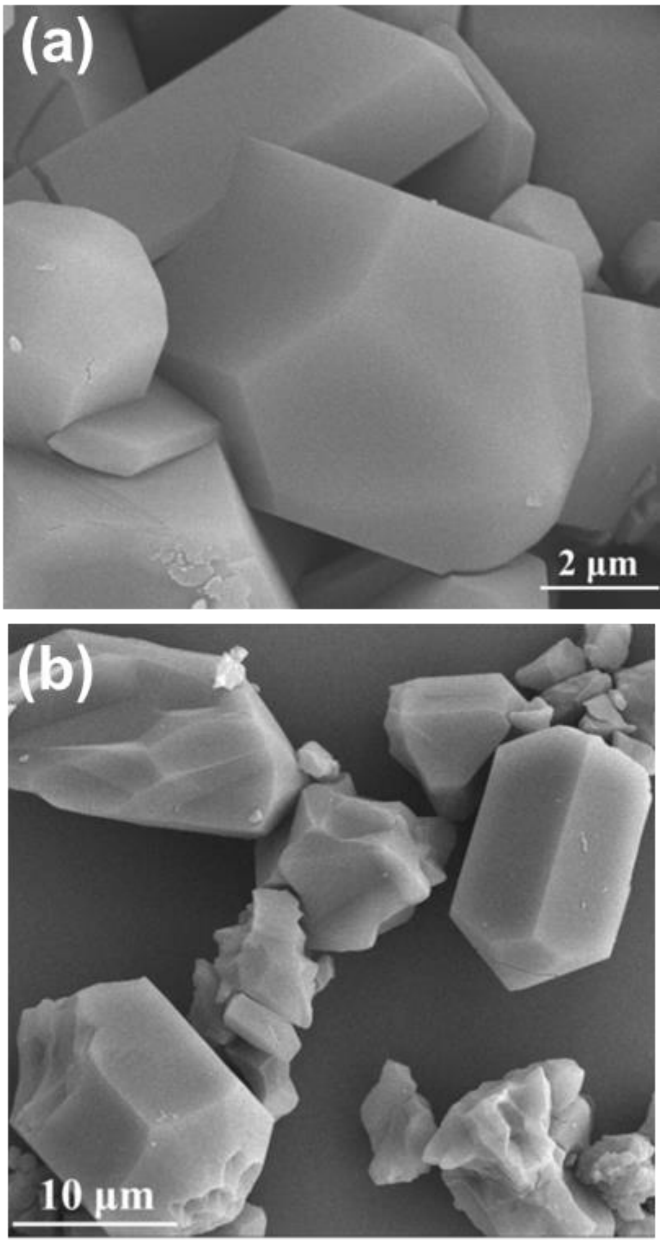

Figure S27 The SEM images of NiTCPE-*pstp* (a) before and (b) after photocatalysis.

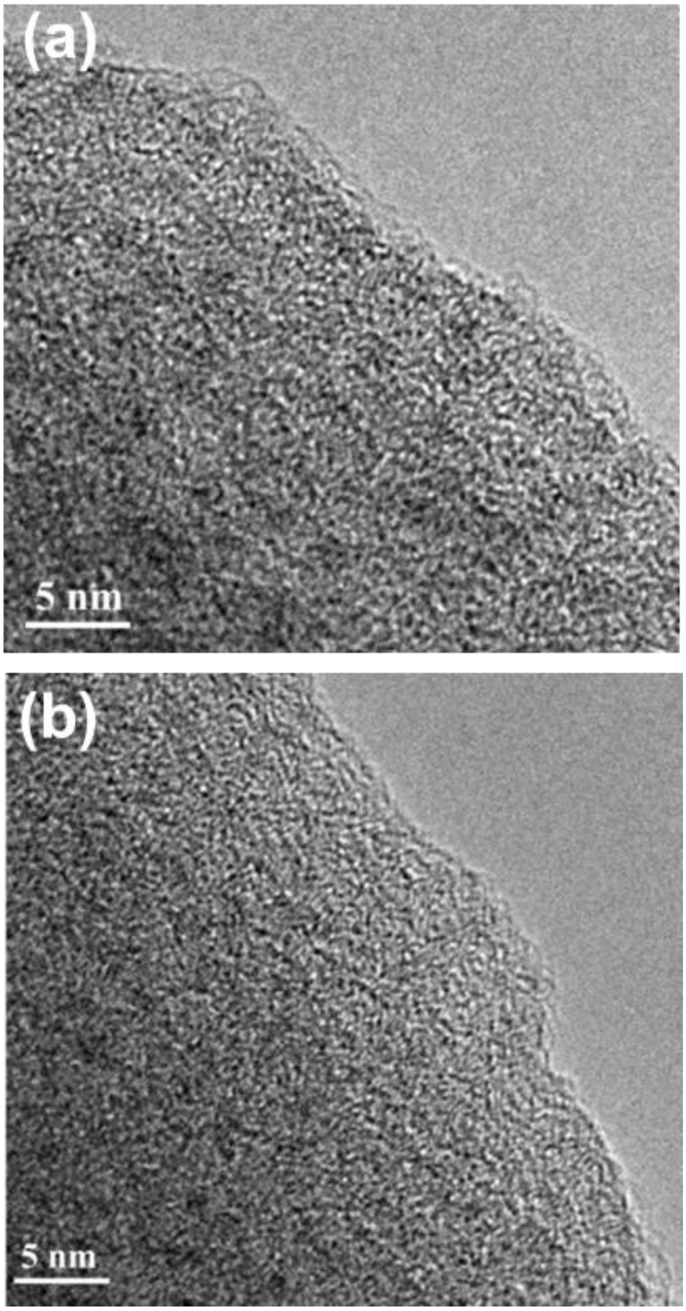

Figure S28 The HR-TEM images of NiTCPE-*pstp* (a) before and (b) after photocatalysis.

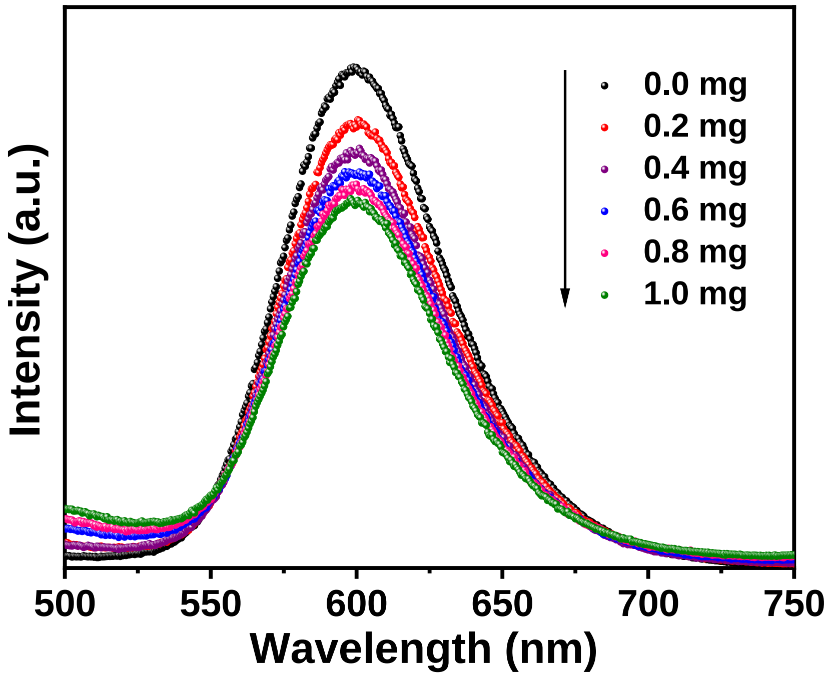

Figure S29 The steady-state PL emission spectra of $[Ru(bpy)_3]Cl_2$ with the addition of NiTCPE-*stp*.

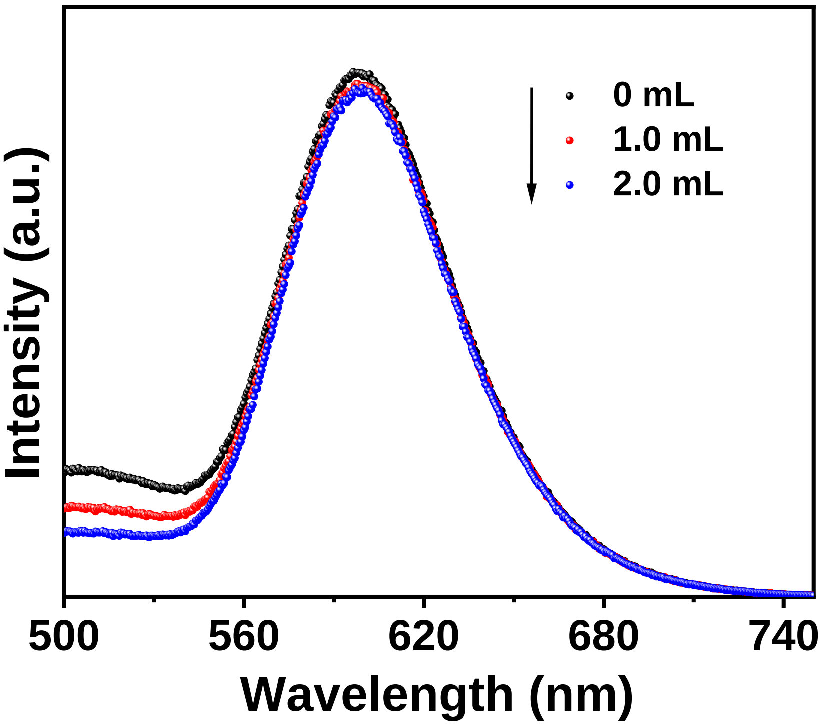

Figure S30 The steady-state spectra of $[Ru(bpy)_3]Cl_2$ upon the addition of increasing amounts of TIPA in the CH_3CN/H_2O solution.

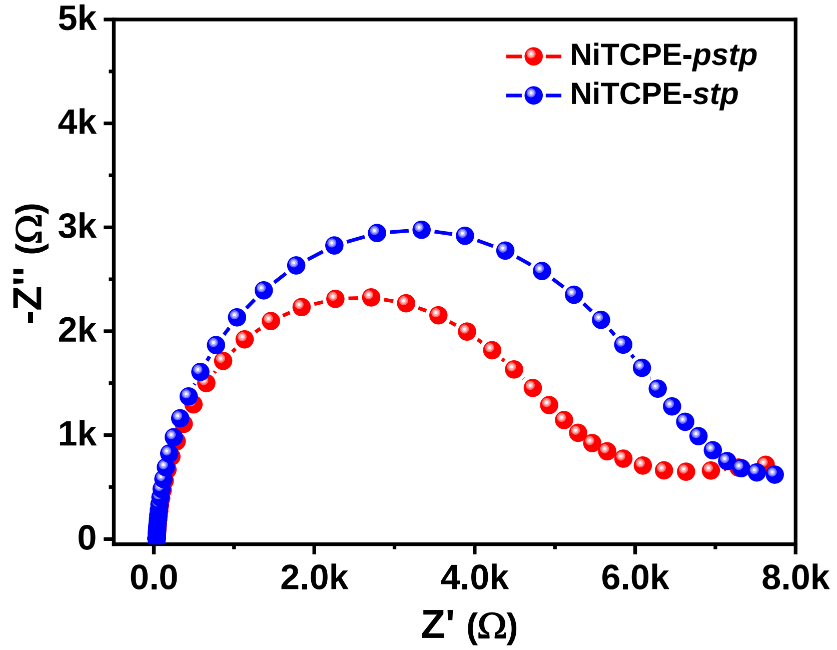

Figure S31 The EIS Nyquist plots for NiTCPE-*stp* and NiTCPE-*pstp*.

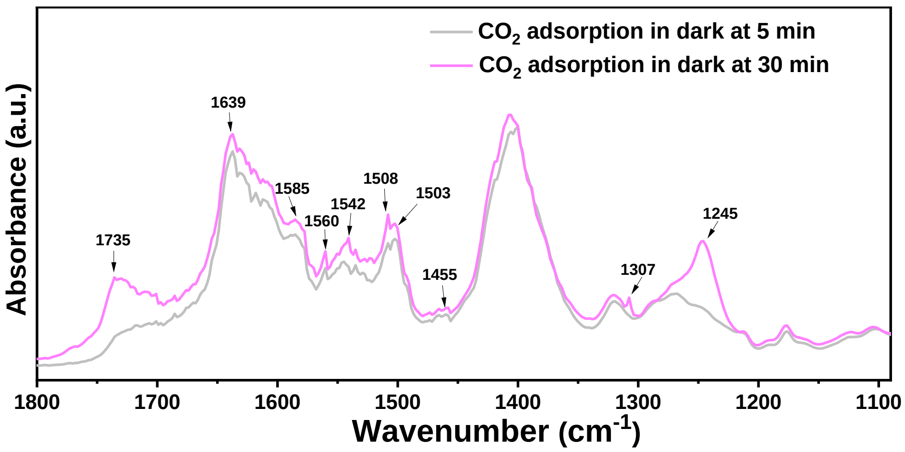

Figure S32 *In situ* DRIFTS for CO_2 adsorption on NiTCPE-*pstp* in darkness at 5 min and 30 min.

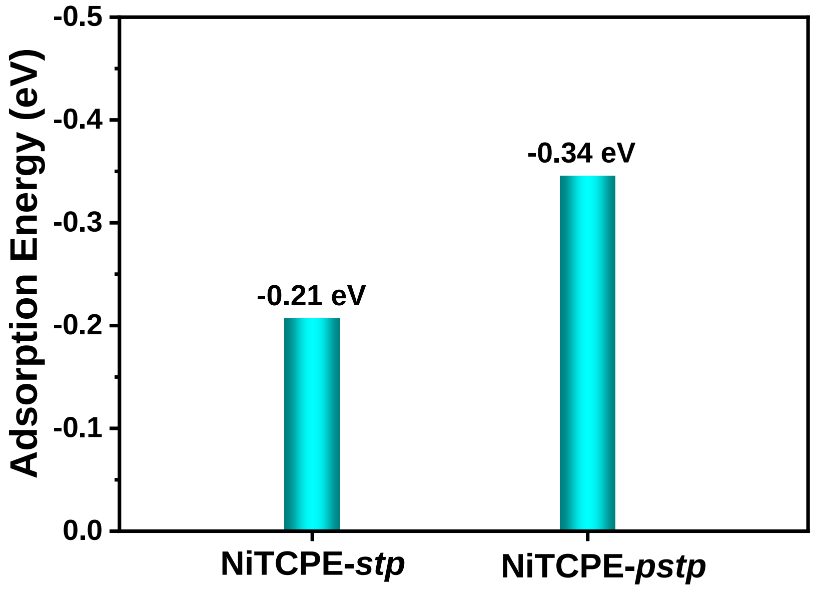

Figure S33 The CO_2 adsorption energy for NiTCPE-*stp* and NiTCPE-*pstp*.

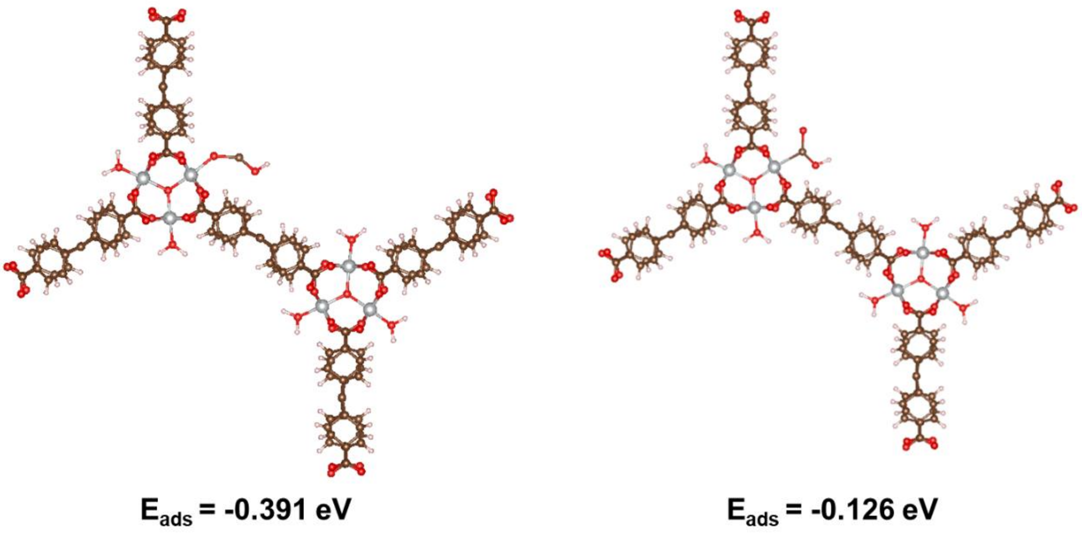

Figure S34 The configuration and adsorption energy of *COOH with C- or O-attaching to the Ni site for NiTCPE-*stp*.

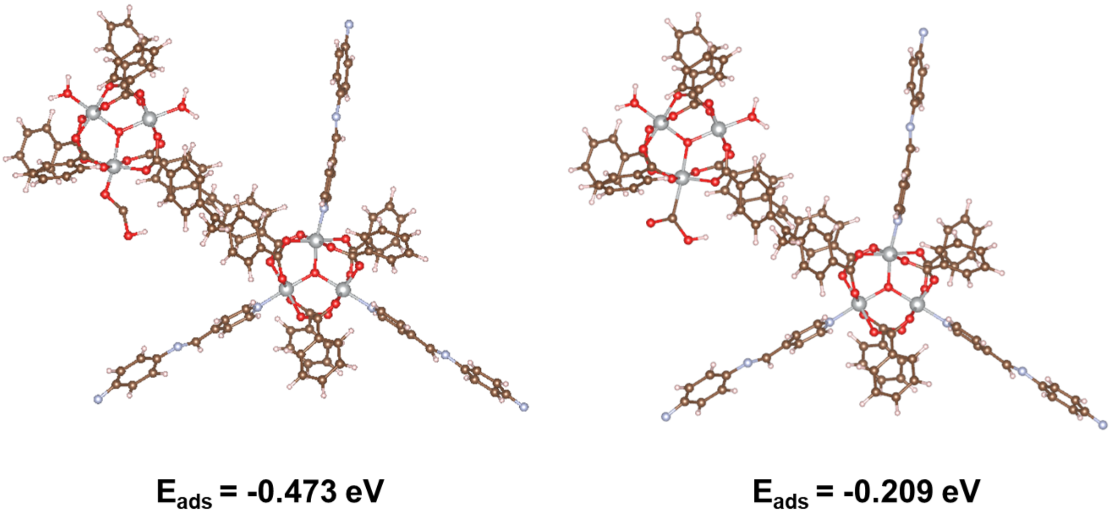

Figure S35 The configuration and adsorption energy of *COOH with C- or O-attaching to the Ni site for NiTCPE-*pstp*.

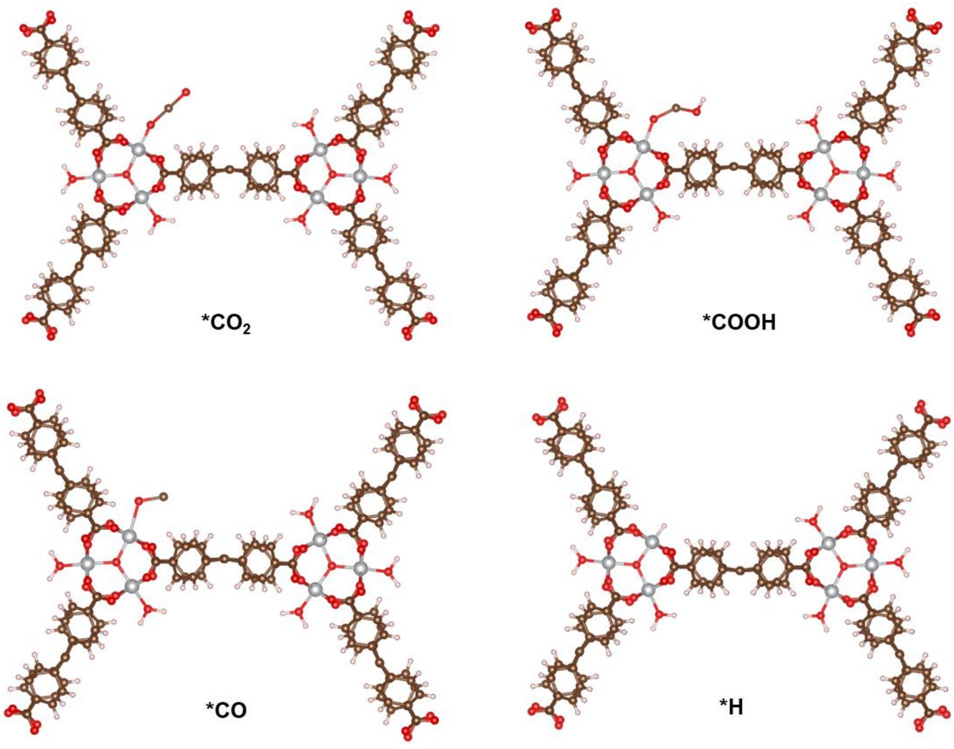

Figure S36 The intermediate structures of photocatalytic CO_2 reduction to CO and H_2 production for NiTCPE-*stp*.

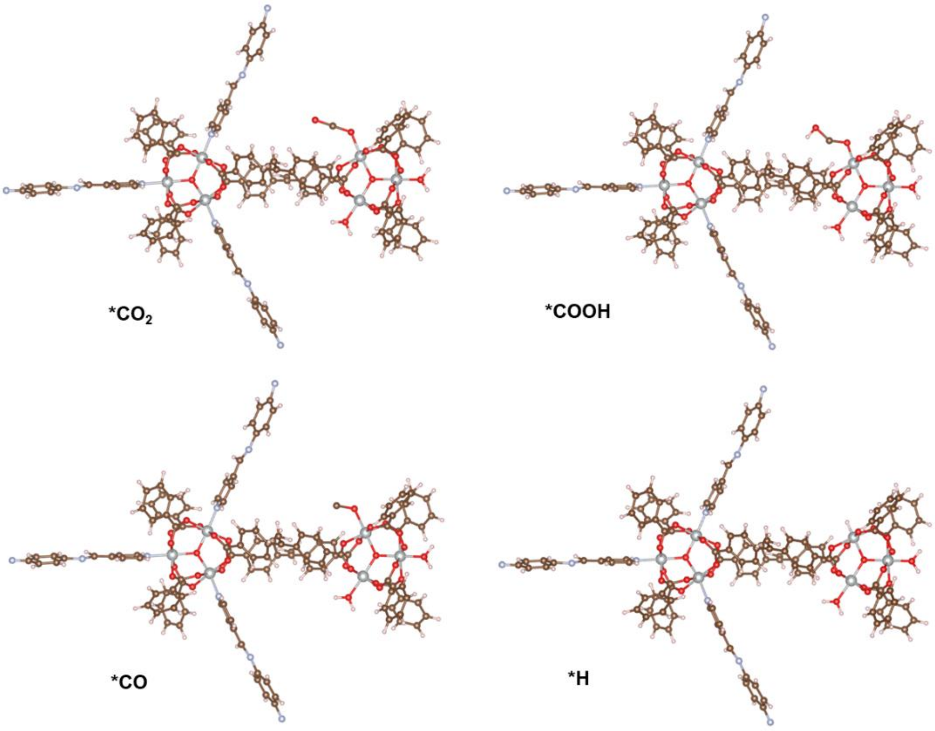

Figure S37 The intermediate structures of photocatalytic CO_2 reduction to CO and H_2 production for NiTCPE-*pstp*.

Table S1 Crystallographic data of TPAPA, NiTCPE-*stp* and NiTCPE-*pstp*.

**Compound**	TPAPA	NiTCPE-*stp*	NiTCPE-*pstp*
Formula	$C_{36}N_7H_{27}$	$Ni_3N_3O_{13}C_{60}H_{39}$	$Ni_6N_7O_{32}C_{126}H_{75}$
Formula weight	557.64	1186.07	2551.19
T (K)	111	293	100
Crystal system	monoclinic	trigonal	hexagonal
Space group	$P2_1/n$	P-3	$P622$
a (Å)	15.4867(6)	26.1017(8)	25.3900(3)
b (Å)	9.7329(2)	26.1017(8)	25.3900(3)
c (Å)	19.8286(6)	11.5431(6)	11.8068(2)
α (°)	90	90	90
β (°)	108.569(4)	90	90
γ (°)	90	120	120
V (Å^3)	2833.17(16)	6810.7(5)	6591.56(19)
Z	4	2	1
D_c.(g cm^{-3})	1.307	0.578	0.643
μ (mm^{-1})	0.404	0.438	2.543
Reflns coll.	21515	46750	30472
Unique reflns	6359	8460	5053
R_{int}	0.0318	0.0836	0.0337
$^aR_1$ [$I \geq 2\sigma(I)$]	0.0419	0.0715	0.0756
$^bwR_2$ (all data)	0.1105	0.2195	0.2255
GOF	1.034	1.052	1.117

$^aR_1 = \sum||F_o| - |F_c||/\sum|F_o|$, $^bwR_2 = [\sum w(F_o^2 - F_c^2)^2/\sum w(F_o^2)^2]^{1/2}$.

Table S2 Selected bond lengths (Å) and angles (°) of NiTCPE-*stp*.

Ni1—O2	1.9935 (3)	Ni1—O4^{iii}	2.051 (3)
Ni1—O6^{i}	2.049 (2)	Ni1—O3	2.051 (3)
Ni1—O5^{ii}	2.048 (3)	Ni1—N1	2.066 (3)
Ni1—N1A	2.046 (4)	O2—Ni1^{iv}	1.9936 (3)
O2—Ni1^{iii}	1.9936 (3)	O6—Ni1^{i}	2.049 (2)
O5—Ni1^{v}	2.048 (3)	O4—Ni1^{iv}	2.051 (3)
O2—Ni1—O6^{i}	93.33 (13)	O6^{i}—Ni1—O4^{iii}	173.36 (11)
O2—Ni1—O5^{ii}	93.06 (13)	O6^{i}—Ni1—O3	88.62 (15)
O2—Ni1—O4^{iii}	93.31 (13)	O6^{i}—Ni1—N1	86.80 (17)
O2—Ni1—O3	93.53 (13)	O5^{ii}—Ni1—O6^{i}	90.97 (15)
O2—Ni1—N1	179.59 (17)	O5^{ii}—Ni1—O4^{iii}	88.77 (15)
O2—Ni1—N1A	179.3 (4)	O5^{ii}—Ni1—O3	173.40 (11)
O5^{ii}—Ni1—N1	86.55 (17)	N1A—Ni1—O6^{i}	86.0 (4)
O4^{iii}—Ni1—O3	90.88 (15)	N1A—Ni1—O5^{ii}	86.9 (4)
O4^{iii}—Ni1—N1	86.56 (17)	N1A—Ni1—O4^{iii}	87.3 (4)
O3—Ni1—N1	86.86 (16)	N1A—Ni1—O3	86.5 (4)
Symmetry codes: (i) -*x*+1, -*y*+2, -*z*+1; (ii) *y*-1, -*x*+*y*, -*z*+1; (iii) -*y*+1, *x*-*y*+1, *z*; (iv) -*x*+*y*, -*x*+1, *z*; (v) *x*-*y*+1, *x*+1, -*z*+1; (vi) -*x*+1, -*y*+2, -*z*+2.			

Table S3 Selected bond lengths (Å) and angles (°) of NiTCPE-*pstp*.

Ni1—O1	1.9739 (7)	Ni1—O3^{iii}	2.076 (3)
Ni1—O2^{i}	2.030 (3)	Ni1—N1	2.100 (8)
Ni1—O2	2.030 (3)	Ni1—O4^{i}	2.124 (11)
Ni1—O3^{ii}	2.076 (3)	Ni1—O4	2.124 (11)
O1—Ni1^{iv}	1.9738 (7)	O3—Ni1^{iv}	2.076 (3)
O1—Ni1^{iii}	1.9738 (7)	O3^{ii}—Ni1—N1	84.2 (5)
O1—Ni1—O2	93.92 (11)	O1—Ni1—O4^{i}	180.00 (2)
O1—Ni1—O2^{i}	93.92 (11)	O1—Ni1—O4	180.00 (2)
O1—Ni1—O3^{ii}	91.47 (11)	O2—Ni1—O2^{i}	172.2 (2)
O1—Ni1—O3^{iii}	91.47 (11)	O2—Ni1—O3^{ii}	90.40 (17)
O1—Ni1—N1	175.4 (6)	O2^{i}—Ni1—N1	84.5 (5)
O2—Ni1—O3^{iii}	89.40 (16)	O2—Ni1—N1	87.7 (5)
O2^{i}—Ni1—O3^{iii}	90.39 (17)	O2^{i}—Ni1—O4^{i}	86.08 (11)
O2^{i}—Ni1—O3^{ii}	89.40 (16)	O2—Ni1—O4	86.08 (11)
O2—Ni1—O4^{i}	86.08 (11)	O3^{iii}—Ni1—O4	88.53 (11)
O2^{i}—Ni1—O4	86.08 (11)	O3^{ii}—Ni1—O4	88.53 (11)
O3^{ii}—Ni1—O3^{iii}	177.1 (2)	O3^{ii}—Ni1—O4^{i}	88.53 (11)
O3^{iii}—Ni1—N1	92.9 (5)	O3^{iii}—Ni1—O4^{i}	88.53 (11)
Symmetry codes: (i) -*y*+1, -*x*+1, -*z*+2; (ii) -*x*+*y*+1, *y*, -*z*+2; (iii) -*y*+1, *x*-*y*, *z*; (iv) -*x*+*y*+1, -*x*+1, *z*; (v) -*x*+1, -*y*, *z*; (vi) *x*-*y*, -*y*, -*z*+1; (vii) *x*-*y*, -*y*, -*z*+2.			

Table S4 The comparison of the photocatalytic performances of reported MOF-based photocatalysts for converting CO_2 to CO under visible light irradiation.

Catalysts	Reaction medium	CO generation rate (μmol g^{-1} h^{-1})	Selectivity (%)	Reference
**Ni_3TCPE-*pstp***	**$Ru(bpy)_3^{2+}$ TIPA**	**3353.8**	**100**	**This work**
TCOF-$MnMo_6$	-	37.25	100	*J Am Chem Soc.*, **2022**, *144*, 1861-1871.
MAF-34-CoRu	-	11.2	100	*J Am Chem Soc.*, **2022**, *144*, 8676-8682.
NNU-55-Ni	$Ru(bpy)_3^{2+}$ TEOA	266.6	81	*Nat Commun.*, **2022**, *13*, 2964.
NNU-55-Ni-NS	$Ru(bpy)_3^{2+}$ TEOA	410.3	81	
π-1-Co	$[Zn(phen)_2L]$ TEOA	494.4	100	*PNAS*, **2022**, *119*, e2118278119.
π-1-Fe	$[Zn(phen)_2L]$ TEOA	396.0	100	
π-1-Ni	$[Zn(phen)_2L]$ TEOA	97.4	100	
TMBen-Perylene	$[Ni(Tpy)_2]^{2+}$ BIH, TEOA	93.0	96	*Angew. Chem. Int. Ed.* **2022**, *61*, e202214142.
H-COF-Ni	$Ru(bpy)_3^{2+}$ TEOA	5694	96	*Adv. Funct. Mater.* **2022**, *32*, 2110694.
Co-MOL@GO	$Ru(phen)_3(PF_6)_3$ TEOA	3133	95	*Nat Commun.*, **2021**, *12*, 813.
HOF-25-Re	$Ru(bpy)_3^{2+}$ TIPA	1448	93	*Angew. Chem. Int. Ed.* **2021**, *60*, 8983 – 8989.
g-C_3N_4-MOLs	2,2'-bpy TEOA	464.1	83.4	*Nano Energy*, **2021**, *80*, 105542.
2D-Co_2TCPE	$Ru(bpy)_3^{2+}$ TEOA	4147	74.4	*Angew. Chem. Int. Ed.* **2020**, *59*, 23588-23592.
2D-Ni_2TCPE	$Ru(bpy)_3^{2+}$ TEOA	3000	97.31	
BIF-29	$Ru(bpy)_3^{2+}$ TEOA	3334	84.1	*Angew. Chem. Int. Ed.* **2019**, *58*, 11752-11756.
Ni-TpBpy	$Ru(bpy)_3^{2+}$ TEOA	811.4	96	*J. Am. Chem. Soc.* **2019**, *141*, 7615-7621.
MOF-Ni	$Ru(bpy)_3^{2+}$ TIPA	371.6	97	*ACS Catal.* **2019**, *9*, 1726-1732.
MOF-Co	$Ru(bpy)_3^{2+}$ TIPA	1140.0	47	
ZrPP-1-Co	TEOA	14	96	*Adv Mater.* **2018**, *30*, 1704388.
$[Ni(bpet)]^{2+}$	$Ru(bpy)_3^{2+}$ BIH	84.5	99	*J. Am. Chem. Soc.* **2017**, *139*, 6538-6541.
MOF-525	TEOA	64.0	91	*Angew. Chem. Int. Ed.* **2016**, *55*, 14310-14314.
MOF-525-Zn	TEOA	111.7	90	
MOF-525-Co	TEOA	200.6	86	
BCN	$Co(bpy)_3^{2+}$ TEOA	93	76.2	*Nat Commun.* **2015**, *6*, 7698.

References

[1] Sheldrick, G. M. *Acta Cryst. A.* **2015,** *71,* 3-8.

[2] Dolomanov, O. V.; Bourhis, L. J.; Gildea, R. J.; Howard, J. A.; Puschmann, H. *J. Appl. Crystallogr.* **2009,** *42,* 339-341.

[3] Kresse, G.; Furthmuller, J., *Comput. Mater. Sci.* **1996,** *6,* 15-50.

[4] Kresse, G.; Furthmuller, J., *Phy. Rev. B* **1996,** *54,* 11169-11186.

[5] Kresse, G.; Hafner, J., *Phys. Rev. B* **1994,** *49,* 14251-14269.

[6] Perdew, J.; Chevary, J. A.; Vosko, S. H.; Jackson, K.; Pederson, M.; Singh, D. J.; Fiolhais, C., *Phys. Rev. B* **1992,** *46,* 6671-6687.

[7] Blochl, P. E., *Phys. Rev. B* **1994,** *50,* 17953-17979.
